# Recent Progress in PDMS-Based Microfluidics Toward Integrated Organ-on-a-Chip Biosensors and Personalized Medicine

**DOI:** 10.3390/bios15020076

**Published:** 2025-01-29

**Authors:** Fahad Alghannam, Mrwan Alayed, Salman Alfihed, Mahmoud A. Sakr, Dhaifallah Almutairi, Naif Alshamrani, Nojoud Al Fayez

**Affiliations:** 1 Microelectronics and Semiconductors Institute, King Abdulaziz City for Science and Technology (KACST), Riyadh 12354, Saudi Arabia; fjakedb@kacst.edu.sa (F.A.); malayed@kacst.edu.sa (M.A.);; 2 Donnelly Centre for Cellular and Biomolecular Research, University of Toronto, Toronto, ON M5S 3E1, Canada; 3 Department of Chemistry, University of Toronto, Toronto, ON M5S 3H6, Canada; 4 Advanced Diagnostics and Therapeutics Institute, Health Sector, King Abdulaziz City for Science and Technology (KACST), Riyadh 12354, Saudi Arabia

**Keywords:** microfluidics, organ-on-a-chip (OoC), PDMS, integration, biosensors, personalized medicine

## Abstract

The organ-on-a-chip (OoC) technology holds significant promise for biosensors and personalized medicine by enabling the creation of miniature, patient-specific models of human organs. This review studies the recent advancements in the application of polydimethylsiloxane (PDMS) microfluidics for OoC purposes. It underscores the main fabrication technologies of PDMS microfluidic systems, such as photolithography, injection molding, hot embossing, and 3D printing. The review also highlights the crucial role of integrated biosensors within OoC platforms. These electrochemical, electrical, and optical sensors, integrated within the microfluidic environment, provide valuable insights into cellular behavior and drug response. Furthermore, the review explores the exciting potential of PDMS-based OoC technology for personalized medicine. OoC devices can forecast drug effectiveness and tailor therapeutic strategies for patients by incorporating patient-derived cells and replicating individual physiological variations, helping the healing process and accelerating recovery. This personalized approach can revolutionize healthcare by offering more precise and efficient treatment options. Understanding OoC fabrication and its applications in biosensors and personalized medicine can play a pivotal role in future implementations of multifunctional OoC biosensors.

## 1. Introduction

Microfluidics, a multidisciplinary field that manipulates fluids at the micron scale, has revolutionized various applications since its emergence in the 1980s [1]. Microfluidic devices offer significant advantages, from inkjet printers to advanced organ-on-a-chip (OoC) and lab-on-a-chip (LoC) technologies. These compact devices, typically just a few centimeters in size, enable the analysis of samples with minimal volume, increased cost efficiency, faster experimentation, and a smaller footprint [2]. Notably, LoC systems integrate complex laboratory processes into miniature platforms, such as fluid transport, mixing, and analysis [3]. Similarly, OoC devices replicate human organ functions, providing new avenues for research into human physiology by mimicking organ functionalities in a controlled microscale environment [4].

The OoC technology represents a paradigm shift in personalized medicine. These devices surpass traditional in vitro models by precisely mimicking human organ functions. By incorporating living cells from a patient’s induced pluripotent stem cells, OoCs offer unparalleled personalization [5]. These platforms replicate organ architecture and physiological conditions, including fluid flow, to simulate individual organ responses. This capability allows researchers to study drug interactions with a patient’s unique genetic makeup, presenting a significant advantage over conventional drug discovery methods that rely on animal models with limited human relevance [4]. For instance, an OoC populated with a patient’s liver cells can predict drug metabolism and identify potential toxicity issues before clinical trials [6]. Despite being in its early stages, OoC technology holds immense promise in studying the effectiveness of drugs on the genetic makeup of the human body. While current models often focus on specific functions, potentially neglecting in vivo complexity, the potential for more human-relevant and patient-specific drug testing is transformative. The OoCs could revolutionize medication development, enhancing efficacy and reducing side [7].

The progress of microfluidics and OoC technology has been driven by significant interdisciplinary collaborations, bridging gaps between biology, engineering, and chemistry. Early milestones in microfabrication techniques, such as photolithography, injection molding, and hot embossing, have laid the foundation for current advancements. Recent innovations, including 3D printing and advanced materials, have further improved the precision and functionality of microfluidic devices [8]. These technological advances have sped up research and broadened commercial applications, from diagnostic tools to therapeutic devices [9]. In the clinical field, microfluidic systems are increasingly used for point-of-care testing, offering quick and accurate diagnostics. The collaboration between academic research and industry has been crucial in translating microfluidic innovations into practical solutions and emphasizing the transformative potential of this technology in scientific research and everyday healthcare [10].

This review article explores the recent advancements in PDMS-based microfluidics for OoC, emphasizing the integration of electrochemical, electrical, and optical biosensors for personalized medicine. It covers the PDMS fabrication process, foundational work, and our contributions, particularly in reviewing the microfabrication of PDMS-based sensors. The integrated biosensors in OoC platforms provide real-time insights into cellular behavior and drug response, which significantly benefit personalized medicine. Devices using patient-derived cells can predict drug effectiveness and tailor therapeutic strategies to individual physiological variations. This study explores the expanding applications of OoCs, including several organs such as (heart, lung, liver, kidney, brain, gut, skin, and multi-organ) on-chip existing structures. Ultimately, the review article presents the future prospects and challenges of OoCs in personalized medicine, aiming to lay the groundwork for future advancements in PDMS-based microfluidics for OoC applications.

## 2. Fabrication Process of PDMS-Based Microfluidics

Microfluidic technologies have gained widespread use across various fields due to their ability to downsize and manipulate fluids [11]. PDMS is a commonly utilized material in microfluidics, and it is favored for creating OoC devices because of its beneficial properties, such as biocompatibility, ease of use, elasticity, optical transparency, and cost-effective fabrication techniques [12]. However, PDMS has some material limitations, such as absorption of organic solvents and hydrophobicity, which is crucial to consider for microfluidic devices. Thus, PDMS’s tendency to adsorb small hydrophobic molecules can lead to significant issues in drug development, mainly when accurate drug concentrations are crucial for bioassays [13]. This absorption can alter drug bioavailability in microfluidic devices like “Organs-on-Chip”, potentially skewing experimental results. One solution to this problem is the application of lipophilic coatings, which have been shown to prevent or reduce small molecule absorption into PDMS [13]. Another approach involves the development of glass-based microfluidic devices, which do not exhibit the same level of compound absorption as PDMS [14]. Additionally, modifying PDMS with smart polymers like PDMS-PEG can decrease nonspecific adsorption while maintaining the material’s biocompatibility [15].

While the hydrophobicity of PDMS impacts fluid flow, surface modifications such as chemical treatment play a role in tailoring it for several applications [16]. Chemical treatments, such as plasma treatment, thermal treatment, and UV curing, offer powerful tools to modify the surface properties of PDMS, adjusting wettability, introducing functional groups, and enhancing biocompatibility [17]. Our prior work on PDMS explores the effects of the curing condition, including time, temperature, and environment, on the optical properties of PDMS at terahertz frequencies. A correlation has been found between curing temperature and the absorption coefficient, in which the absorption coefficient is increased due to the higher cross-linked network formed at the higher temperature. Thus, the underlying physics of the curing process is important for implementing PDMS-based microfluidics for OoC devices [18]. Furthermore, blending PDMS with nanocomposites can enhance mechanical strength and chemical stability [19]. Overall, these treatment methods unlock the full potential of PDMS for OoC and other microfluidic applications, expanding its capabilities and performance [1,20]. This section reviews the main techniques for fabricating PDMS-based microfluidic devices, including photolithography, injection molding, hot embossing, and 3D printing. Each method offers distinct advantages and challenges, which are discussed below.

### 2.1. Photolithography

**Figure 1:**
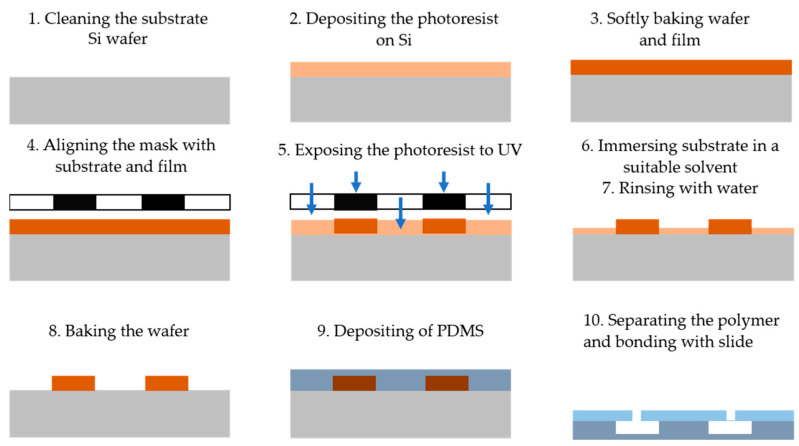
The main steps of the photolithography technique for microfluidic device fabrication including cleaning the substrate wafer, depositing the photoresist on a silicon substrate, baking the wafer into the oven, aligning the photomask containing the pattern with the substrate, exposing the photoresist to UV, immersing the coated substrate in a solvent, rinsing the substrate with DI water, baking the wafer, depositing PDMS, and separating the polymer from the substrate and bonded with flat slide.

Photolithography is a technique that utilizes light to define patterns on a photosensitive material, which is then used to fabricate microfluidic devices [21]. The process is outlined in Figure 1 and consists of the following steps: (1) cleaning the substrate wafer (e.g., Si), (2) depositing a layer of photoresist onto the silicon substrate, (3) placing the wafer in an oven for soft baking, (4) aligning a photomask containing the desired microstructure pattern with the substrate and photoresist, (5) exposing the photoresist to ultraviolet (UV) light through the mask to crosslink it in the desired pattern, (6) immersing and stirring the coated substrate in a suitable solvent (the developer) to remove the exposed portions of the photoresist for positive resist or the non-exposed portions for negative resist, (7) rinsing the substrate with deionized (DI) water, (8) baking the wafer to remove residual solvent and water, (9) depositing a PDMS layer on the substrate to transfer the pattern onto the PDMS surface, and (10) separating the PDMS from the substrate and bonding it to a flat slide with inlet and outlet ports to complete the microfluidic system [22].

Photolithography offers numerous advantages, particularly in terms of high precision and resolution. It allows for creating patterns with nanometer-scale detail on various surfaces [23]. The alignment process enhances the accuracy and resolution of microfluidic device fabrication. Additionally, photolithography is relatively cost-effective, making it an affordable option for producing various microsystems. However, the technique does have limitations. One challenge is using UV light, which can present issues in 3D microfluidic device fabrication. The process also requires skilled operators, particularly when creating complex structures. Furthermore, the cost of producing advanced microfluidic models is high, as it necessitates specialized equipment and cleanroom facilities. Environmental concerns related to the chemical fabrication process must also be considered.

### 2.2. Injection Molding

**Figure 2:**
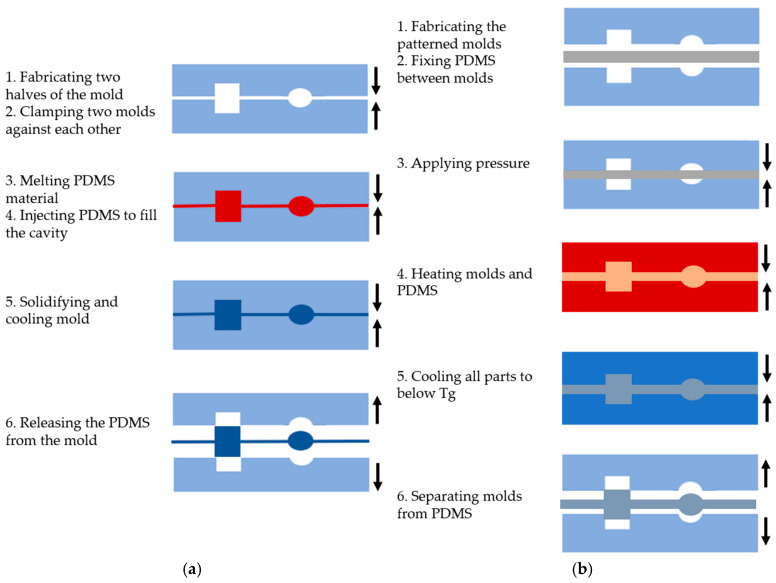
The main steps of PDMS-based microfluidic system fabrication using (**a**) the injection molding method; (**b**) the hot embossing methods.

Injection molding is a widely used technique for processing PDMS and creating various microfluidic system shapes [24,25]. This method is performed in six basic steps, as shown in Figure 2a: (1) creating two halves of a mold with the desired topographic pattern, (2) clamping the two mold halves against each other in a machine press, (3) melting the PDMS to turn it into a liquid, (4) injecting the molten polymer into the heated mold cavity to fill it completely, (5) allowing the molds to solidify and cool below the glass transition temperature (Tg) of the PDMS, and (6) releasing the solidified PDMS by separating the mold halves [24].

Injection molding has gained popularity in microfluidic system fabrication due to several advantages. One of the key benefits is high replication accuracy, as high-volume production can maintain consistent properties due to the molten nature of the polymer during the injection. This method allows for the creation of complex microstructures, such as systems used in 3D cell culture [26]. Moreover, the process enables the integration of other fluidic components (e.g., connectors and pumping tools). Various materials, particularly polymers, can be used for microfabrication, and the process is cost-effective and fast. Injection molding can also produce microstructures with excellent filling without trapping air bubbles, which is essential for microfluidic applications. However, there are some limitations to consider. The cooling time of the polymer is critical, as it can lead to thermal stress and defect formation if the cooling process is too rapid [26]. Only thermoplastic materials are suitable for manufacturing complex 3D devices, which imposes material restrictions [27].

### 2.3. Hot Embossing

The hot embossing method involves transferring mold features to a PDMS substrate under high temperature and pressure, similar to injection molding, as depicted in Figure 2b. The process includes the following steps: (1) creating the desired pattern on a solid material, such as silicon, to form the molds, (2) placing the PDMS between the molds, (3) applying pressure range of kPa, (4) heating the molds for a period of time, (5) cooling all parts to below the glass transition temperature (Tg) of the PDMS, and (6) carefully separating the molds from the PDMS [28].

Hot embossing offers several advantages over injection molding. It enables the fabrication of microfluidic devices at the nanoscale (sub-50 nm), which are highly sought after for biological fluid applications [29]. The technique is also cost-effective, making it more accessible to researchers and commercially viable for microfluidic companies. In contrast to injection molding, the PDMS flows only a short distance, reducing material stress and minimizing shrinkage [30]. This method allows for the creation of small features and high aspect ratios due to the low pressure, low flow rate, and controlled cooling rates applied during the process. However, there are some limitations. Hot embossing can be more expensive and time-consuming when 3D molds are required. The process involves both heating and cooling the polymer and molds, which takes longer than injection molding. Typical processing times for hot-embossed parts range from 4 to 30 min. Additionally, non-uniform temperature distribution can lead to distortion in the mold due to internal stresses [31].

### 2.4. 3D Printing

3D printing is a fabrication technique that creates objects layer by layer, with each layer being added on top of the previous one. This process is utilized to produce three-dimensional microfluidic devices, and several technologies have been developed to construct the object layers [32]. The primary 3D printing methods include fused deposition modeling (FDM) [33], stereolithography (SL) [34], selective laser melting (SLM) [35], and Multi-Jet Modeling (MJM) [36]. In FDM, the lowest layer is created by melting a thermoplastic filament (such as polystyrene), extruding it through a nozzle, and allowing it to cool and solidify. The process is then repeated for each subsequent layer, gradually building the 3D structure [37]. On the other hand, stereolithography (SL) utilizes photosensitive thermoset polymers to create 3D microfluidic devices. The LED or UV light is focused onto a vat of photopolymer resin to shape and solidify the resin according to a computer-aided design, starting with the bottom layer of the desired structure [34]. Selective Laser Melting (SLM) is an additive manufacturing process in which a focused laser beam scans a layer of PDMS powder, selectively fusing the material based on the pattern provided. The laser heats the material, triggering chemical reactions that fuse and solidify the layer [35]. In Multi-Jet Modeling (MJM), tiny photopolymer droplets are ejected from multiple inkjet print heads onto a removable platform. Each droplet is cured immediately by UV light, forming the desired layer. By repeating this process, the entire 3D structure is gradually created.

In recent years, 3D printing techniques have gained significant attention for fabricating microfluidic devices due to their many advantages. These methods allow for the creation of parts and assemblies from multiple materials with varying physical and mechanical properties in a single-build process [31]. 3D printing also enables high-throughput production of microfluidic devices compared to other techniques that require clean room facilities, and 3D printing offers a more cost-effective fabrication process [38]. Additionally, recent advancements have improved the resolution of 3D-printed microstructures to the scale of tens of micrometers [31]. One benefit of 3D printing is the ability to easily adjust features by simply modifying the device design in the software. This flexibility allows for the production of complex, functional 3D shapes tailored to user specifications, including flow-regulating components and robust connection ports [38]. Furthermore, 3D is generally more affordable, faster, and easier to use than other microfabrication methods. However, there are some limitations to 3D printing for microfluidics. For devices with high-resolution features (below 100 μm), removing resin and support material from the structure can be challenging [31]. Also, few available transparent materials for microfluidic fabrication can be used in 3D printing, which hinders the utilization of 3D printing in many applications [24]. Additionally, multilayer structures may fracture due to insufficient fusion between adjacent layers [30]. Finally, some low-resolution 3D printers can reduce surface profiles on the printed microstructures.

## 3. Integrated Sensors with OoC Platform

In this section, we will mention three main categories of integrated sensors and explain the types of each category in the following subsections. The three categories of integrated sensors are electrochemical, electrical, and optical.

### 3.1. Integrated Electrochemical Sensors

Electrochemical devices are versatile in transforming interactions between analytes and electrodes into measurable electrical signals [39,40]. These signals, typically current or potential differences, enable detecting a broad range of analytes, from oxygen to specific ions crucial for pH measurement. This core functionality lays the groundwork for biosensor development by incorporating a biochemical recognition element [41]. Notably, potentiometric and amperometric techniques offer straightforward and practical implementation for biosensing applications. At their heart, electrochemical sensors excel at converting selective (bio)chemical interactions into electrical outputs. Their inherent simplicity, reliability, and amenability to miniaturization and cost-effective production make them ideal candidates for integration with microfluidic devices. This synergy extends their applications significantly within the biological and biomedical realms [42]. In the field of bioelectrochemistry, there are four primary classifications of electrochemical biosensors: amperometric, potentiometric, voltammetric, and impedimetric. Each type is characterized by its unique method of electrical measurement for signal transduction, effectively converting biological recognition events into measurable electrical signals, including current, potential, or variations in impedance. The versatility of electrochemical sensors highlights their immense potential for future advancements in biochemistry, biotechnology, and biomedical engineering [43,44,45].

#### 3.1.1. Amperometric Sensors

It functions by applying a constant potential to the working electrode, inducing electron transfer reactions with target analytes in the surrounding medium. The resulting current is then measured and correlated to the analyte concentration using Faraday’s Law [46]. This current reaches a steady-state value, and a critical step involves distinguishing between capacitive and faradaic currents to quantify the electroactive species accurately. Chronoamperometry is a prominent technique within this framework, where the current response is monitored over time at a constant potential. Amperometric sensors have gained significant traction in recent years, particularly within biomedical diagnostics, due to their appealing attributes—high sensitivity, accuracy, stability, and portability [47]. These sensors essentially leverage a fixed electrode potential to trigger electron transfer at the electrode surface with target molecules, enabling direct detection and quantification based on the resulting current [48,49].

Within electroanalytical chemistry, amperometry is vital in continuously monitoring currents produced by biochemical reactions involving the oxidation or reduction of electroactive species [50]. A classic illustration of this principle is the Clark oxygen electrode, an amperometric biosensor. A precisely controlled potential applied to a platinum working electrode facilitates oxygen reduction. A silver-silver chloride (Ag/AgCl) reference electrode completes the circuit. Crucially, the current generated by the sensor exhibits a direct proportionality to the concentration of oxygen present. This linear relationship arises because the current depends on the rate of oxygen reduction at the electrode surface. As oxygen concentration increases, the reaction rate accelerates, leading to a correspondingly amplified current. This proportionality allows the sensor to assess oxygen levels quantitatively via current measurement [46]. However, it is essential to distinguish amperometry from voltammetry. While amperometry focuses on a single, constant potential for current measurement, voltammetry employs a dynamic approach. Here, the current is monitored as the applied potential is systematically varied. Voltammetry’s analytical power hinges on the linear relationship between peak current and analyte concentration within a specific potential range. Higher peak currents directly correspond to greater concentrations of the target electroactive species in solution. This dependence allows researchers to quantify the analyte based on the measured peak current [46].

Despite their impressive sensitivity, a significant hurdle is the inherent lack of electroactivity in many protein analytes. These proteins struggle to readily donate or accept electrons at the working electrode, hindering direct detection. To overcome this limitation, amperometric devices often employ mediated electrochemistry. Here, a mediator molecule acts as a bridge, facilitating electron transfer between the protein analyte and the electrode. While this strategy introduces additional complexity, it offers a significant benefit—signal amplification. This allows amperometric sensors to achieve much lower detection limits than potentiometric devices. However, it is crucial to acknowledge a potential trade-off. Introducing a mediator can introduce additional complications and potentially compromise the selectivity of the measurement, meaning the sensor might struggle to distinguish the target protein from other interfering species [46].

#### 3.1.2. Potentiometric Sensors

**Figure 3:**
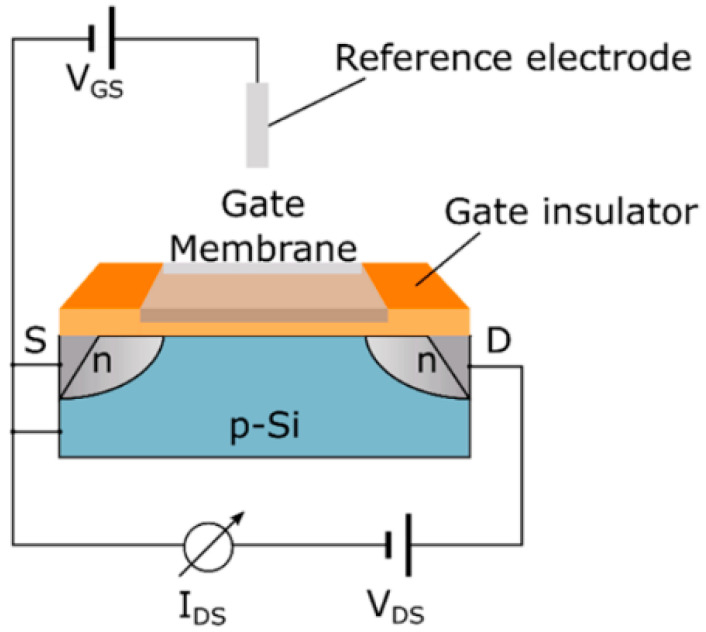
An ion-sensitive field-effect transistor (ISFET) functions by establishing a voltage across its source and drain electrodes to detect variations in current. The gate region is equipped with an ion-selective membrane or biological receptors, which modulate the current flowing between the drain and source [43]. Adapted with permission from reference [43].

The assessment of various chemical species, including ions like sodium and potassium, as well as pH levels and carbon dioxide concentrations, is fundamental in many analytical contexts. These sensors measure the possible difference between an indicator electrode and a reference electrode. There are two principal types of indicator electrodes in use: Metal oxide (MOx) sensors and ion-sensitive field-effect transistors (ISFETs), representing key chemical sensing technologies. MOx sensors apply the Nernst equation, which links the measured potential difference to the solution’s pH, demonstrating how pH influences the surface potential and electrical properties of the metal oxide material. In contrast, ISFETs are based on silicon substrates and feature an ion-sensitive membrane integrated with a field-effect transistor (FET) along with a reference electrode, as depicted in Figure 3 [43]. In FET, the application of voltage between the source and drain terminals induces a conductive channel beneath the gate electrode, which regulates the current flow as a function of the applied voltage. This electric field influences the current at the gate and is further affected by charged species present above it. By integrating molecular receptors or an ion-sensitive membrane on the gate region, ISFETs can distinguish specific ions or charged biomolecules based on their interactions with the membrane [43,46].

In electroanalytical chemistry, potentiometry reigns supreme for extracting information on ionic activity within electrochemical reactions [51]. This methodology systematically examines the voltage difference between the active and reference electrodes within a uniquely constructed electrochemical cell. Notably, this measurement occurs under conditions that minimize or prevent current flow across the system. This allows for precisely determining the potential, free from the influence of ongoing electrochemical reactions. The linchpin of this approach is the Nernst equation, which forges a crucial link between the concentration of ions and the potential measured during a potentiometric experiment. Direct potentiometry harnesses the power of this equation to quantify the concentration of target analyte ions directly. Notably, ion-selective electrodes (ISEs) stand as the champions of potentiometry, boasting some of the lowest detection limits currently achievable, reaching analyte-specific ranges between 10^−8^ and 10^−11^ M [52]. A key strength of potentiometric sensors lies in their exceptional ability to analyze low concentrations within minuscule sample volumes. This advantage stems from their ideal characteristic of leaving the sample chemically unaltered. However, while these techniques offer impressive shallow detection limits, a key limitation lies in their applicability. These methods are often restricted to a specific range of ions, unfortunately excluding analytes of significant environmental and biological interest, such as nickel, manganese, mercury, and arsenate ions. This selectivity constraint necessitates further development to broaden the scope of detectable analytes and fully unlock the potential of these shallow detection limit methods [51].

#### 3.1.3. Voltammetric Sensors

It functions by analyzing the system’s response to a continuously changing electrical potential. This variation in potential, applied to an electrochemical cell, elicits a corresponding current reaction. A voltammogram characteristic curve is generated by plotting this current against the applied potential [53]. This versatile technique encompasses various methods, each with its strengths for specific applications, including differential pulse, stripping, square wave, and cyclic voltammetry. Conductive polymers (CPs), due to their sensitivity to analyte-induced changes, offer a valuable approach to developing voltammetric sensors. Voltammetric biosensors, a specialized type, leverage these principles to glean information about target analytes. They measure the current response of a biological recognition element (receptor) immobilized on the electrode surface as the potential varies. Within a defined linear potential range, the measured peak current directly correlates to the analyte concentration in the solution. This dependence allows quantifying the target analyte based on the measured peak current, offering a powerful analytical tool for biosensing applications. Traditionally, working electrodes in voltammetric sensors were constructed from mercury, carbon materials, or inert metals [54]. However, mercury has been phased out due to toxicity concerns and handling difficulties. While established carbon materials like nanotubes, glassy carbon, graphene, and diamond remain valuable, their high cost can be limiting. Carbon paste electrodes have emerged as attractive alternatives in recent years due to their compatibility with mass-production techniques like screen printing, making them a viable option for large-scale applications. Screen-printed electrodes offer another attractive option due to their robustness, miniaturization, and suitability for commercial applications. Most commercially available electrochemical sensors, such as glucose sensors, utilize screen-printed electrodes. This technology holds immense potential for expansion beyond the glucose sensor market. The speed, operational simplicity, real-time analysis capabilities, and the possibility of miniaturization for portable use make voltammetric sensors promising candidates for various diagnostic methods [48,55,56].

#### 3.1.4. Impedimetric Sensors

Operate by measuring the electrical impedance of a system in response to biomolecular interactions. This can involve applying a direct current and measuring resistance (applicable to resistive, conductometric, or capacitive sensors) or introducing an alternating current at various frequencies and analyzing the resulting impedance spectrum [57]. Extensive research has yielded diverse strategies for designing impedimetric biosensors [58]. One approach involves creating surface functional groups (carboxyl, amino, etc.) on glass or gold using salinization or alkanethiol monolayer formation. These functional groups then serve as anchor points for covalent attachment of biomolecules like enzymes or antibodies. Alternatively, biomolecules can be physically entrapped using electrochemically deposited polymers, gel coatings, or layer-by-layer assembly techniques. While conductometric methods within impedimetric devices can assess the conductivity of materials and solutions, their application in biosensing faces limitations. The variable ionic background of clinical samples can obscure the target signal, and measuring minute conductivity changes in highly conductive media is challenging. Impedimetric biosensors offer a more robust solution. They directly monitor changes in the electrode’s conductance upon immobilizing biorecognition elements like enzymes or antibody-antigen pairs onto the surface, providing a more sensitive and specific signal for biosensing applications [44,46].

#### 3.1.5. Evaluation of Integrated Electrochemical Sensors

Integrating various electrochemical sensor technologies—namely amperometric, potentiometric, voltammetric, and impedimetric sensors—plays a pivotal role in enhancing the functionality of organ-on-a-chip platforms and advancing personalized medicine. Each sensor type presents distinct advantages and challenges: amperometric sensors are distinguished by their high sensitivity and real-time monitoring capabilities, albeit with potential stability concerns [47]; potentiometric sensors offer simplicity and low power requirements, which are particularly beneficial for wearable applications, although they may exhibit slower response times [46,47]; voltammetric sensors excel in specificity and sensitivity, yet their dependence on precise electrode materials can impede miniaturization efforts [47,54]; meanwhile, impedimetric sensors facilitate label-free detection and seamless integration with nanomaterials, despite the necessity for complex signal interpretation and meticulous surface preparation [57]. Collectively, these sensor modalities underscore the importance of careful optimization and adaptation to specific applications, ultimately enhancing the precision, sensitivity, and integration of organ-on-chip systems in diagnostics and personalized medicine.

### 3.2. Integrated Electrical Sensors

Incorporating electrical sensors within PDMS-based OoC platforms significantly advances disease modeling and drug discovery efforts. These integrated sensors enable real-time, in-situ monitoring of crucial tissue microphysiological parameters. This enhances the model’s fidelity and offers a deeper understanding of complex biological processes. An illustrative example of such a platform is the silicon-polymer hybrid OoC device equipped with charge sensors and recording microelectrodes. This novel design exploits the natural biocompatibility and optical transparency of polymers at the sensing interface, simultaneously leveraging silicon’s outstanding electrical properties and its capability for integrating active electronic functionalities. This synergistic approach allows for sensitive, multi-modal monitoring of the microenvironment, paving the way for more robust and translatable pre-clinical assessments [59,60].

Sensor fabrication employs a BiCMOS-based cleanroom process at the wafer level, yielding a population of nominally identical microchips with dimensions optimized for seamless integration with liquid handling components, such as 3D-printed microfluidic wells and holders. A crucial aspect of this process involves the development of custom-designed printed circuit boards (PCBs). These PCBs ensure compatibility with commercially available microelectrode array (MEA) readout systems, enabling efficient liquid management within the sensing areas [59,61]. Furthermore, there is a burgeoning interest in the synergistic integration of diverse sensing technologies, encompassing pH, oxygen levels, and additional parameters, to comprehensively understand cellular behavior and responses in real-time [62,63]. This trend toward incorporating multiple sensors within multi-organ platforms is an emerging field that aims to broaden the utility of OoC devices across various research domains [63,64]. Diverse electrical sensors are pivotal in OoC platforms, monitoring and analyzing multiple physiological parameters in real-time. These sensors enhance functionality by providing continuous data acquisition, facilitating a deeper understanding of cellular behavior, tissue responses, and environmental conditions within the OoC microenvironment [43,44,65,66]. Commonly employed electrical sensors in OoC platforms include:

#### 3.2.1. Electrical Sensors

Within the domain of OoC, electrical sensors reign supreme as the most prevalent sensing methodology. This ubiquity can be attributed to two key factors: their inherent simplicity of integration and the established expertness in microelectronics, facilitating the seamless incorporation of reduced electrodes. Notably, these electrodes empower the measurement of critical parameters encompassing cellular and physical properties. On the cellular front, they illuminate details of tight junction formation within barrier epithelia and provide morphological insights. Regarding physical properties, they enable the analysis of strain, which is precious in monitoring the contractile activity of cardiac cells [43].

#### 3.2.2. Cell Impedance Sensors

**Figure 4:**
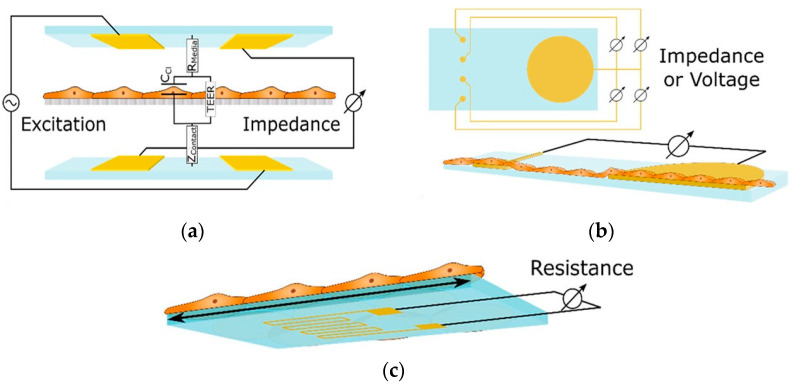
Shows three essential sensing modalities in OoCs. (**a**) Trans-epithelial/Endothelial Electrical Resistance (TEER) quantifies barrier integrity via resistance across a cultured cell membrane; (**b**) Patterned electrodes with two different techniques, field potential, and Electrical Cell-Substrate Impedance Sensing (ECIS) sensors, analyze the behavior of cells in specific areas. Field potential sensors capture electrical activity (voltage) outside cells, while ECIS gauges cell properties (impedance) at the interface; (**c**) A strain gauge affixed to a conductive element within the device quantifies the deformation induced by cellular activity. These techniques offer comprehensive insights into various cellular functions within OoCs [43]. Adapted with permission from reference [43].

These sensors measure alterations in impedance within microfluidic pathways, enabling the assessment of cellular adhesion, proliferation, and morphology. The trans-epithelial/endothelial electrical resistance (TEER) reigns as the dominant electrical sensing method. Measured across a cell-cultured semipermeable membrane (Figure 4a), TEER quantifies barrier integrity, with high values reflecting tight junction formation. This guarantees the accurate in vivo recapitulation of permeability investigations. Additionally, TEER, with proper modeling, can reveal other aspects like cellular differentiation. Impedance spectra fitting or single-frequency measurements provide normalized cell resistance (Rcells, Ω cm^2^). Electrical cell-substrate impedance sensing (ECIS) offers a localized analysis of cell behavior via patterned electrodes (Figure 4b). This method extends beyond tight junctions to evaluate cellular functions [43].

Mermoud et al. have created an innovative microimpedance tomography (MITO) system designed to be integrated with lung-on-a-chip platforms. This framework aims to tackle the difficulties associated with conventional TEER assessments in lung-on-chip models, which are influenced by the rhythmic motions of the barrier that occur during the respiratory process. The MITO system employs three coplanar impedance electrodes integrated into a flexible PCB to assess the lung alveolar barrier from a distance of 1 mm, facilitating movement. This advancement addresses the constraints of earlier systems, which had electrodes positioned significantly closer to the barrier. The MITO system can detect alterations in both electrical and mechanical properties of the barrier, including those induced by respiratory movements. It can even detect slight variations in the mechanical strain due to differences in cell density. This technology has the potential to provide valuable physiological data on biological processes and disease development in the lungs, making it a promising tool for drug discovery and other applications [45,67].

#### 3.2.3. Extracellular Field Potential Sensors

Multielectrode arrays (MEAs), intricately woven with isolated microelectrodes, examine the electrophysiological secrets of Organs-on-Chip. Directly culturing electrically active cells (think neurons and cardiomyocytes) on their surface, MEAs capture the subtle shifts in extracellular field potential as cells depolarize and repolarize. By monitoring voltage spikes exceeding a set threshold, these arrays unveil the symphony of action potential firing with high spatial resolution, thanks to the rapid decay of electrical signals with distance. This versatile platform simultaneously measures individual cells’ high-frequency whispers and coupled activity’s low-frequency hum, reflecting cellular and organ-level physiology. By investigating the spatial and temporal characteristics of these signals with parameters such as the duration of field potentials, intervals between peaks, and conduction velocity, MEAs elucidate the complex nature of electrophysiological responses in cultured cell systems [43].

**Figure 5:**
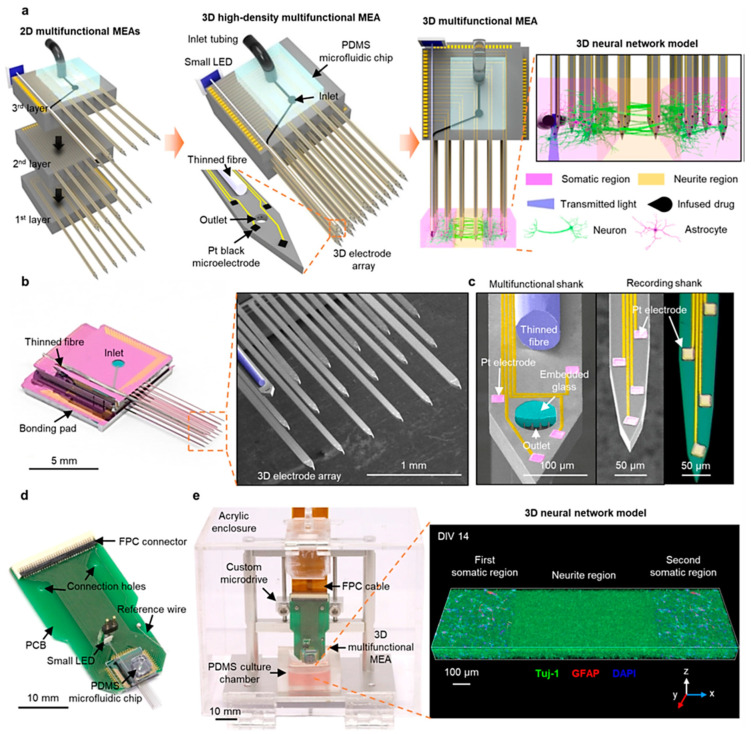
Illustrate the 3D microelectrode array’s high-density, multifunctional capabilities encompassing its design principles, fabrication techniques, packaging strategies, and integration methods. (**a**) illustrates the assembly process, showcasing individual 2D MEAs before stacking, the final assembled 3D MEA with a microfluidic interface, and its application to a compartmentalized 3D neural network model; (**b**,**c**) present high-resolution images of the 3D MEA and its components, including the multifunctional shank with its embedded features; (**d**) Displays the packaged 3D MEA with a light-emitting diode and a flexible connector. Finally, (**e**) Depicts the complete 3D MEA system integrated with a Microdrive and a culture chamber and showcases a 3D rendered image of a populated neural network within the system [68]. Adapted with permission from reference [68].

Shin et al. presented a novel three-dimensional high-density MEA, which combines optical stimulation techniques, microfluidic systems for drug delivery, and high-density recording functionalities. This MEA features 18 microfabricated shanks, including a multifunctional one with embedded optical fiber and microfluidic channels, as shown in Figure 5a–c, achieving dense recording coverage (114 sites/mm³) within 3D neural tissues. For in vitro experiments, they packaged the 3D MEA by bonding it to a custom PCB for electrical connections, a Microdrive for manipulation, and a PDMS microfluidic chip for drug delivery, see Figure 5d,e. Furthermore, a light-emitting diode (LED) was attached to the fiber for simplified operation, shown in Figure 5d, and the recording capability was enhanced by electrodepositing platinum black onto the electrodes for increased surface area. 3D MEA design offers significant potential to revolutionize in vitro investigations of neural circuits. This technology facilitates the acquisition of precise measurements for synaptic latencies and the capability for localized network modulations. This combined functionality promises to unveil a deeper understanding of neural network dynamics in a controlled vitro environment [68].

Beyond Shin et al. recent high-density 3D MEA, advancements in MEA technology encompass diverse strategies to enhance performance. These advancements include integration with impedance spectroscopy within microfluidic devices, disposable MEAs, and silicon-polymer hybrid multi-modal platforms. These innovations have empowered researchers to achieve higher precision and accuracy in neural activity measurement and analysis, significantly contributing to the development of neuroprosthetics and related fields [59,68,69].

#### 3.2.4. Strain Sensors

**Figure 6:**
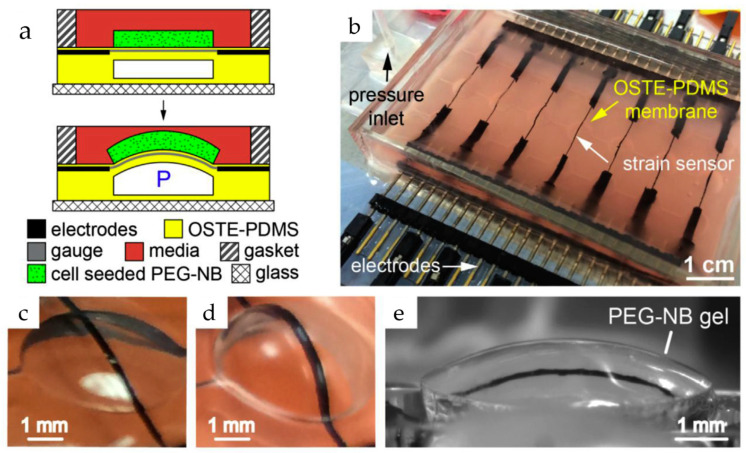
The device combines a flexible sheet (membrane) with built-in carbon nanotube (CNT) sensors to apply 3D pressure and measure how cells respond in gels at the same time. (**a**) Gels-containing cells are attached to the membrane using a particular chemical reaction. This allows the membrane to squeeze and stretch the gel repeatedly. Tiny sensors made of carbon nanotubes constantly track how much the membrane bends (strain) in response to pressure (P); (**b**) An image of the device with the built-in CNT sensors. Wires for electrical signals are hidden within the membrane for insulation; (**c**) shows a single section of the membrane with the sensor at rest; (**d**,**e**) shows the membrane bending with and without the gel attached [70]. Adapted with permission from reference [71].

Strain gauges offer a powerful tool for deciphering the mechanical forces exerted by cells within OoCs. These sensors are usually made up of an inactivated conductive meander that is securely attached to a substrate (see Figure 6). They can measure small deformations, such as the bending of a membrane used in cell culture, which may be related to various cellular activities, including the contraction of cardiac tissue. The principle relies on the change in resistance experienced by the meander as it elongates or compresses along its conductive paths. However, strain gauges are often integrated into Wheatstone bridges due to the small magnitude of this change relative to the overall resistance. This configuration converts the measured resistance change into a differential measurement, significantly enhancing accuracy [43].

Liu et al. developed a new microdevice platform for studying tissue development. This innovative platform combines real-time stiffness monitoring with the ability to apply mechanical forces (stretching or compression) to 3D cell-laden hydrogels. Such an approach could overcome a major hurdle in current methods, which only analyze tissues at the end and miss crucial dynamic changes. Furthermore, they successfully used the platform to track stiffness variations in hydrogels containing stem cells under different conditions. The microdevice in such a platform builds upon their existing bulging membrane technology. This method features 3D hydrogels containing the target tissues bonded to these membranes, which are then stretched. Embedded carbon nanotube sensors measure the resulting deflection, with the amount of deflection reflecting tissue stiffness. Changes in sensor resistance (|ΔR/R0|) due to strain are used to calculate the tissue’s elasticity [70]. The fabrication process in such a platform involves creating microchannel-laden membranes from a specific PDMS mixture and bonding them to a glass base. A CNT-PDMS blend is then screen-printed onto the membranes to create strain sensors. Furthermore, calibration and assembly with a cell culture chamber complete the device. The researchers further explored integrating tunable poly (ethylene glycol)-norbornene (PEG-NB) hydrogels containing cells onto the membranes. These hydrogels were synthesized and covalently bonded using thiol-ene click chemistry. By incorporating specific peptides, they achieved controllable degradation and adhesion properties. Finally, mesenchymal stem cells were incorporated into a PEG-NB solution to create cell-laden hydrogels. A microfluidic technique with stencils and a photomask formed these hydrogels with embedded cells directly on the membrane, resulting in a covalently bound and cell-encapsulating interface [70].

#### 3.2.5. Evaluation of Integrated Electrical Sensors

Ultimately, integrating advanced electrical sensors—encompassing Electrical Sensors, Cell Impedance Sensors, Extracellular Field Potential Sensors, and Strain Sensors—into organ-on-a-chip (OoC) platforms signifies a pivotal advancement in personalized medicine. These sensors collectively enhance the capability to perform high-resolution monitoring and facilitate real-time analyses of cellular dynamics. However, challenges related to material compatibility, system miniaturization, and environmental variability persist [63,66]. Notably, while Cell Impedance Sensors and Extracellular Field Potential Sensors provide invaluable insights into cell behavior and electrical activity, their efficacy is often limited by electrode fouling and noise susceptibility [59,64,65,68]. Furthermore, Strain Sensors contribute crucial biomechanical data yet demand further optimization to reduce invasiveness and improve durability [44,70]. Comparative studies underscore the necessity for innovations in material science, alongside enhancements in sensitivity and biocompatibility, to fully harness the potential of these technologies. Ultimately, the convergence of these advancements positions OoCs as transformative platforms for developing predictive, patient-specific diagnostics and therapeutic testing, marking a significant step forward in pursuing individualized medical care.

### 3.3. Integrated Optical Sensors

Optical sensing techniques have been developed rapidly in recent years and have shown great promise as an essential piece in LoC systems such as PDMS-based OoC devices. Monitoring OoC models presents a significant challenge, where continuous and precise observation of the biological and physiological parameters within the chip in real-time is highly demanded [9].

Optical sensors have several attractive features, making them a great alternative to traditional sensing strategies. One of the main benefits is the easy fabrication, integration, calibration, and validation of optical sensors within OoC systems compared to other monitor types, such as electrode sensing that requires advanced microfabrication tools [71,72,73]. Miniaturization, low manufacturing costs, and commercially available optical sensors highly contributed to the possibility of placing sensors inside a microfluidic system and achieving more experiments to improve the interpretation of results [74,75]. In addition, optical detection can provide information on various critical parameters in the cell culture matrix, such as metabolic activity, cellular pathology, tissue viability, cell growth, and cell-to-cell interactions [73]. Another main advantage of optical integration in OoC systems is that they are contactless with the detecting element and the sensor, where the signals can be transmitted in microfluidic materials, such as glass, or through a window. Contactless measurement gives this sensor significant potential for multi-parameter systems without requiring a reference element compared to an electrochemical sensor [43]. One of the advantages of optical sensing integration is that measurement does not consume oxygen because of the independence of redox reactions, which is important in the microliter volumes applied in OoC systems [62]. Optical monitoring can be achieved in real-time by covering a broad spectrum of sensing techniques and long-term analysis where cells are generally not disrupted during experiments [76]. The temperature stability of optical sensors up to 180 °C during measurement could help increase the result resolution [43].

Optical sensor integration in OoC systems still needs some development and more research to overcome some disadvantages. In the beginning, optical sensing techniques require a dye or labeled substrate for visualizing the compound in the cell culture microfluidic, which requires knowledge of material chemistry and optics to determine polymer materials and indicator dyes. Additionally, for the long-standing stability of the optical detector, photodegradation, called bleaching of the sensor, was observed where some dyes degrade and lose their brightness after interaction with light, besides the limited range of substance concentrations [43]. These dyes may leach into the cell culture matrix and deteriorate the sensor sensitivity over time. Another limitation of optical sensors that highly affects the measurement performance is the optical length through the sample where the sensitivity reduction occurs as described by the Beer-Lambert law [76]. The limitation of absorbance measurement was also observed, resulting from the background fluorescence of some culture medium components [76]. To overcome these limitations, many efforts have been invested. Different dyes and matrices were developed in the case of a dye or labeled substrate of visualization that increases the possibilities of using optical sensing integration of the OoC chip [75]. To avoid photodegradation, dyes that have high photostability can be employed by reducing the light exposure of the sensing components [77]. The range expansion of matrix concentrations can be achieved by color development, such as adding absorbable phenol red [9]. For the elimination of leaching, covalently bonding between larger molecules or trapped particles and medium can be made [77].

In general, microfluidic devices, such as the OoC systems, with integrated optical sensors comprise two parts: a transparent microfluidic material, e.g., PDMS, already discussed above, and an optical detection system. Most integrated optical sensors contain a detector element, a transducer transducing the recognition event output signals, and a signal processing device converting output optical signals into appropriate readings [78]. The detector elements can be determined depending on the optical and measurement principle and monitor format. For example, intensity-based sensors typically require an excitation source such as a light emitting diode (LED) or lamp and sensitive indicator dye to the analyte of interest and matrix chip [76].

**Figure 7:**
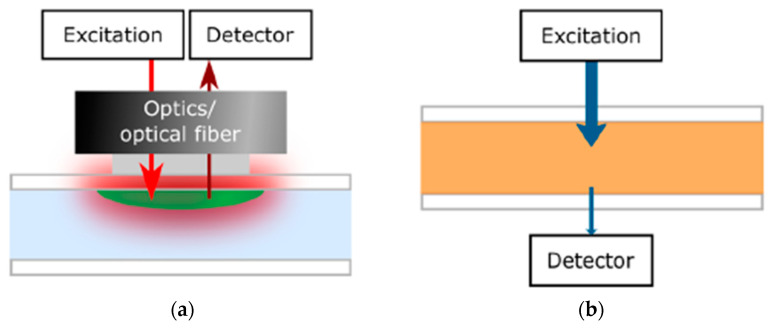
Diagram illustration of the optical monitor setup on the basis of (**a**) luminescence where the detector is located on the same side of the excitation light source and (**b**) Absorption measurements [43]. Adapted with permission from reference [43].

Before the integration of the optical sensing element into the OoC system, the optical sensing principle should be recognized beside multiple biomarkers of organs. Optical sensors depend on detecting changes in optical proprieties of cell or culture microenvironment, including absorbance, fluorescence intensity, refractive index, and scattering. One of the main monitors successfully integrated into a flexible OoC structure was the luminescence sensor [79]. In this sensor technique, molecules are excited by a light source at a wavelength absorbed by the indicator dye, resulting in emission spectra captured via the detector that is usually mounted on the same side of the light source, as shown in Figure 7a [43]. Different measurement types of luminescent sensors can be achieved, such as lifetime, luminescent intensity, and temperature [80]. Lifetime-based measurement, which depends on the average time the fluorophore stays in the excited level before releasing a photon, has higher contrast and suppression of background signals than intensity-based sensors. Another integrated optical monitor system into the OoC device was an absorption sensor depending on the analyte concentration correlated to the dye’s molar absorption based on the Beer−Lambert law, as illustrated in Figure 7b [43].

**Figure 8:**
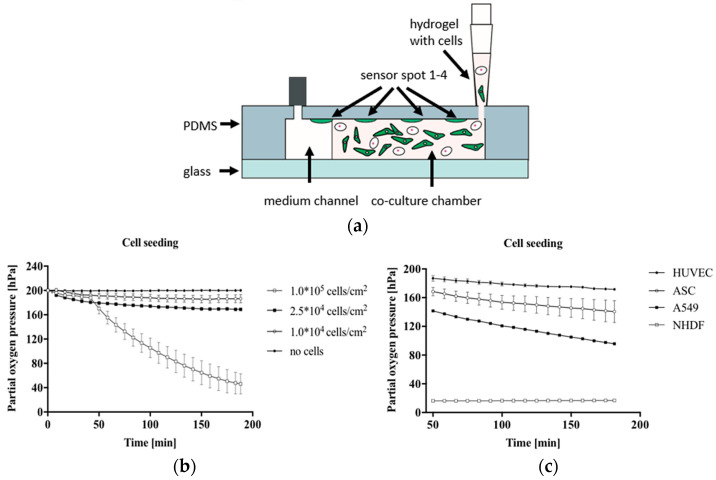
In (**a**) The cross-section of microfluidic chips during cell and hydrogel seeding into a gas-permeable 3D chip; (**b**) The effect of A549 cell density on the consumption of oxygen was recorded during 3 h of cell seeding into the chip; (**c**) The impact of cell type on the pressure of partial oxygen during the feeding process of cells [81]. Adapted with permission from reference [81].

Several recent attempts have been made to integrate optical sensors successfully into the OoC system using different-based measurements. Zirath et al. presented the incorporation of an optical oxygen meter into a flexible microfluidic PDMS using optical fibers to measure the oxygen level in a cell culture medium [81]. The number of effects of A549 lung cells on oxygen consumption and the influence of cell types, including A549 lung epithelial, human umbilical vein endothelial cells (HUVEC) endothelial, normal human dermal fibroblasts (NHDF), and adipose-derived stem cells (ASC) stem cells on oxygen levels, were investigated. Figure 8a illustrates the hydrogel and cell seeding into a gas-permeable 3D culture microfluidic chip with integrated oxygen detector spots. The results show a variation in oxygen consumption for increasing the numbers of cells after 10 min of cell seeding, indicating more oxygen requirements in the case of larger lung cell numbers, as shown in Figure 8b. At the same density of 2.5 × 10^4^ cells/cm^2^, the impact of cell types on oxygen level was clearly observed, where the highest oxygen consumption was recorded to be 183 ± 0.4 hPa for NHDF fibroblast cells compared to the lowest of 28.4 ± 0.4 hPa for HUVEC endothelial cells, as illustrated in Figure 8c. The outcome of this study supports the ability to integrate microparticle-based sensor spots for estimating cellular oxygen consumption.

**Figure 9:**
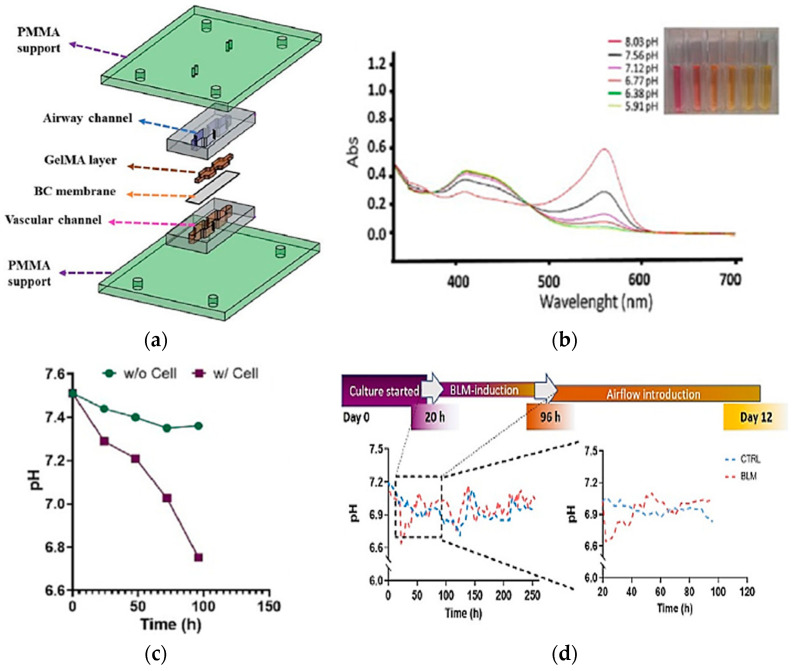
(**a**) Design of sensor integrated into microfluidic to observe major features of cells in cell culture matrix; (**b**) The changes of absorbance caused by the pH changes vs. the UV-VIS wavelength scan; (**c**) The pH changes recorded based on the time with and without the cell’s presence in culture media; (**d**) Time-dependent pH level changes in control and BLM-induced PF model and zoomed pH changes during 96 h of incubation [82]. Adapted with permission from reference [82].

Real-time pH measurements are important for examining cell and organ functions in the chip. The integration of absorbance-based sensing into LoC systems was one of the attempts to monitor the pH value of the culture media. Saygili et al. designed a pH sensor containing an optical sensor (3 mm light-dependent resistor) and LEDs at wavelengths of 430 and 560 nm for monitoring real-time pH changes in a microfluidic chip during the physiological and pathological changes resulting from bleomycin induction [82]. The biomimetic microfluidic device was built using a matching design of PDMS channels sandwiched between two PMMA layers, as shown in Figure 9a. The result shows the absorbance changes because of the pH changes, as shown in Figure 9b, and the decrease of pH value over time with/without cell in culture media, as presented in Figure 9c. The same approach was observed when the cells were hatched for 12 days in DMEM-F12 media as a control. Conversely, the descents and ascents of pH level were observed in the bleomycin (BLM)-induced model, particularly within the first 96 h, as shown in Figure 9d. This study presents the possibility of optical sensor integration into an OoC system for pH change measurement with high reservoir volume and dynamic flow.

**Figure 10:**
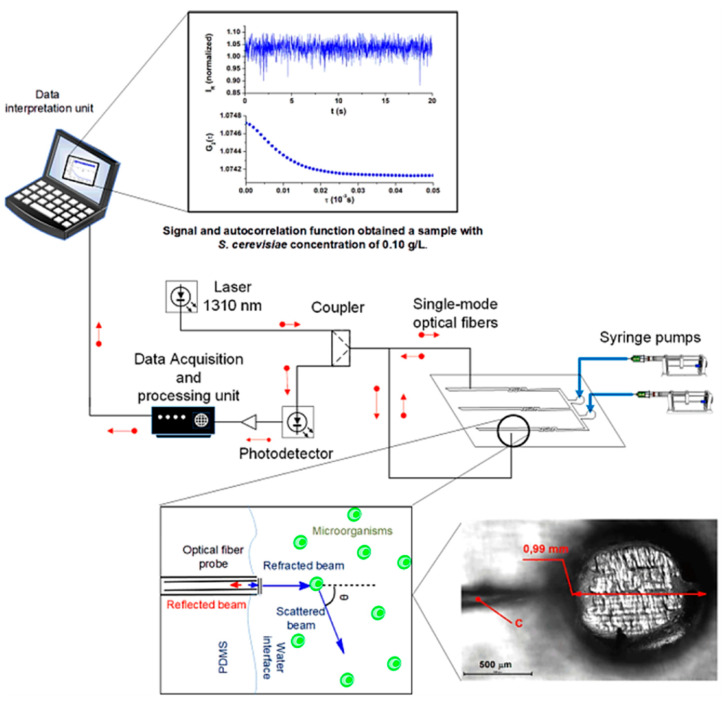
The setup of the PDMS microfluidic system integrated into an optical fiber sensor containing SMF, 1310 nm laser system, and photodetector for the monitoring Saccharomyces cerevisiae cells concentration depending on the light scattering inside the microchamber [83]. Adapted with permission from reference [83].

Optical sensors based on quasi-elastic light scattering have been successfully integrated with microfluidic devices for cellular growth analysis by Soares’s team [83]. The group fabricated a PDMS reversible microfluidic bioreactor comprising channels and perfusion chambers equipped with single-mode optical fibers (SMF) inserted inside the chambers during PDMS curing over 3D-printed molds. Experimentally, as shown in Figure 10, a portion of the 1310 nm laser beam launched through an SMF is transmitted to the medium containing Saccharomyces cerevisiae ATCC 7754 cells, and the other portion is reflected to the same fiber with intensity depending on the total number of cell growth. The reflected light is directed to a measurement system, a photodetector, to monitor the microbial kinetics and cell concentration.

**Figure 11:**
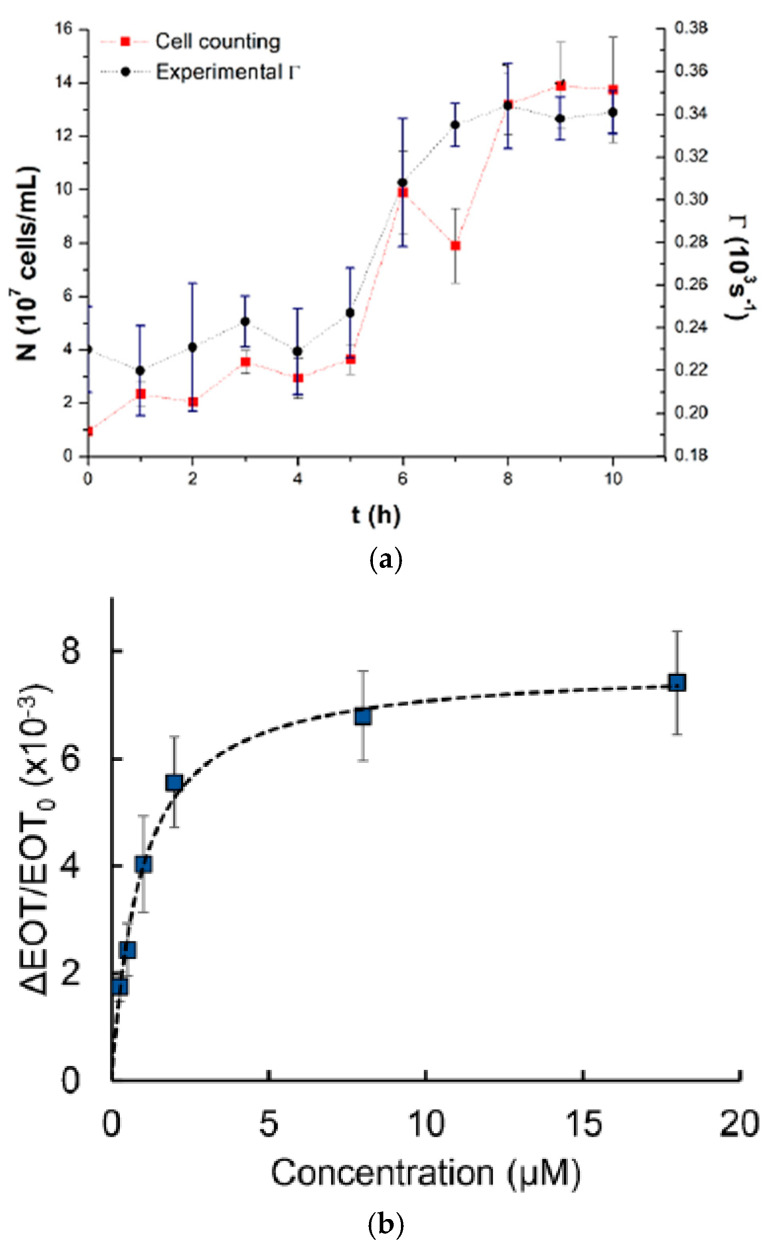
(**a**) The direct relationship between the cell counting (cells/ mL) curve and the average decay rate Γm vs. fermentation time using an optical fiber sensor with chamber procedure [83]; (**b**) Averaged relative EOT variations at different concentrations of the 60 kDa his-tagged protein from the family of Arabinanase [84]. Adapted with permission from references [83,84].

The result shows that a bioreactor can be utilized for the direct and online observing of the Saccharomyces cerevisiae fermentation process where both cell concentration (number of cells/mL) and decay rate values (Γm) were obtained as a function of time as presented in Figure 11a. Another team worked on real-time monitoring of 60 kDa his-tagged protein detection using a label-free porous silicon optical biosensor [84]. Graham et al. designed PDMS channels at dimensions of 500 μm in width and 200 μm in height based on a 3D-printed polyacrylate model integrated with PSi to monitor the PSi’s reflectivity changes. The microfluidic device has been successfully used to measure the averaged relative effective optical thickness (EOT) differences for the protein at the range of concentration between 0.25 and 18 μM, as presented in Figure 11b. The super achievement of the 3D-printed fluidic system integrated aptasensor confirms the ability to detect various target cells.

To summarize, the integration of optical sensors within organ-on-a-chip (OoC) platforms signifies a pivotal enhancement in the realms of personalized medicine and organ-specific biosensing. These advanced systems markedly improve the predictive accuracy of disease models while enabling real-time monitoring of physiological parameters. Recent investigations have demonstrated the effective incorporation of these sensors in lung-on-chip and liver-on-chip models, allowing for the assessment of crucial metrics such as oxygen levels, pH, and cellular metabolism through the application of optical fluorescence and electrochemical sensing methodologies [74,80,84]. A primary advantage of these integrated optical sensors lies in their ability to provide continuous and non-invasive data, which facilitates in-depth analysis of dynamic cellular responses [81]. However, challenges remain, including biofouling, signal stability, and the complexities of incorporating multifunctional sensors within microfluidic architectures [43]. For example, while fluorescence-based sensors offer significant sensitivity and scalability, their performance may be compromised by background interference in intricate biological environment chips [76]. Conversely, optical oxygen sensors integrated into liver organoids demonstrate potential for precise monitoring of metabolic flux, yet their successful application necessitates the development of advanced microfabrication techniques [43].

Combining these technologies with microfluidics and tissue engineering advancements can significantly enhance their potential. Improved reliability may facilitate the development of high-throughput personalized treatments and more effective disease modeling [43,62,72]. To further advance these technologies, enhancements in 3D printing resolution and adopting smoother bonding methods, such as UV-curable adhesives, are imperative. Additionally, addressing issues such as non-specific adsorption in PDMS-based systems will be crucial. Material science and sensor integration advances can mitigate variability and enhance device scalability. These innovations are poised to refine the accuracy and reliability of OoC platforms, thereby broadening their application within personalized medicine. Ultimately, this could lead to improved drug testing methodologies, enhanced disease modeling, and the development of tailored therapeutic interventions [83,84].

Ultimately, the potential of THz spectroscopy integration with microfluidic platforms as a THz-biosensor has been explored in our recent work, emphasizing its potential for label-free and reagent-free sensing. We highlighted the importance of selecting appropriate materials and structures that impact THz absorption and overall performance. While non-polymeric materials like quartz are effective, polymers such as PDMS are becoming viable alternatives due to their cost advantages. The works also discussed pioneering biological studies demonstrating improved spectroscopic performance with these integrated platforms, particularly in reducing water absorption. Our findings suggest significant potential for further advancements in this novel integration [85].

## 4. Integrated OoC Platforms for Personalized Medicine

In the wake of the Human Genome Project’s completion, genomics has experienced a surge driven by technological advancements [86]. The streamlined generation, analysis, and interpretation of genomic data have increased efficiency and cost-effectiveness [87]. Despite these strides, integrating these advancements into clinical practices has been gradual. At the core of this transformation lies personalized medicine, an evolving healthcare discipline rooted in recognizing the uniqueness of each individual [88]. Traditional medical practices are characterized by a ‘one-size-fits-all’ model and transition to a more refined and individualized methodology [89,90]. Personalized medicine embodies a holistic approach, integrating clinical, genetic, genomic, and environmental information to individualize patient care across the health-to-disease spectrum. Leveraging molecular understanding, this approach optimizes preventive healthcare and introduces targeted drug therapies in early disease stages, aiming to tailor medical care for maximal individual outcomes [91,92]. Technologies like mass spectrometry, high-throughput sequencing, imaging, and microfluidics enable intricate measurements, revealing molecular and cellular variations between individuals. Analytics fused with these technologies usher in a new era of precision medicine, guiding tailored patient management strategies [93].

OoCs have garnered significant attention beyond academic circles. Acknowledged as one of the top budding technologies in 2016 by the World Economic Forum, the OoC addresses the imperative need for human-like testing systems in the pharmaceutical, cosmetic, food, and chemical industries [94]. This acknowledgment underscores the potential of OoCs to revolutionize testing methodologies, replacing or complementing traditional approaches such as animal testing with more humanized in vitro alternatives. OoCs have evolved into a transformative translational science paradigm, poised to significantly impact drug discovery, disease modeling, and toxicity assessment [7]. By providing a more accurate representation of human diseases and responses to drugs, OoCs offer novel tools for researchers and contribute to the ongoing era of tissue chip research [95]. However, challenges and limitations exist, necessitating a thorough understanding of the context of use for OoC platforms to ensure their continued development and application. This section will probe into the advancements of OoC technology, mainly focusing on platforms based on PDMS and integrated with diverse biosensors. These OoCs emulate human organ functions at microscale levels and are instrumental in revolutionizing biomedical research and pharmaceutical testing. Specifically, we explore PDMS-based models simulating the complexities of lung, liver, brain, and gut tissues individually and integrated multiorgan chips. Each OoC incorporates sophisticated biosensors tailored to monitor metabolic activities, tissue barrier functions, and electromechanical properties within these microphysiological systems. This comprehensive approach enhances our understanding of organ-level responses to drugs and diseases. Also, it underscores the potential of OoCs to replace traditional experimental models with more accurate, human-relevant alternatives in biomedical applications and personalized medicine.

### 4.1. Integrated PDMS-Based OoC Sensors

This section will review the recent work in the integrated OoC systems, including lung, liver, heart, brain, and gut-on-a-chips, and multi-OoC systems. At the end of Section 4, Table 1 will summarize the biosensor types, targets, applications, and advantages for all discussed Organ-on-a-Chip.

#### 4.1.1. Lung-on-a-Chip

**Figure 12:**
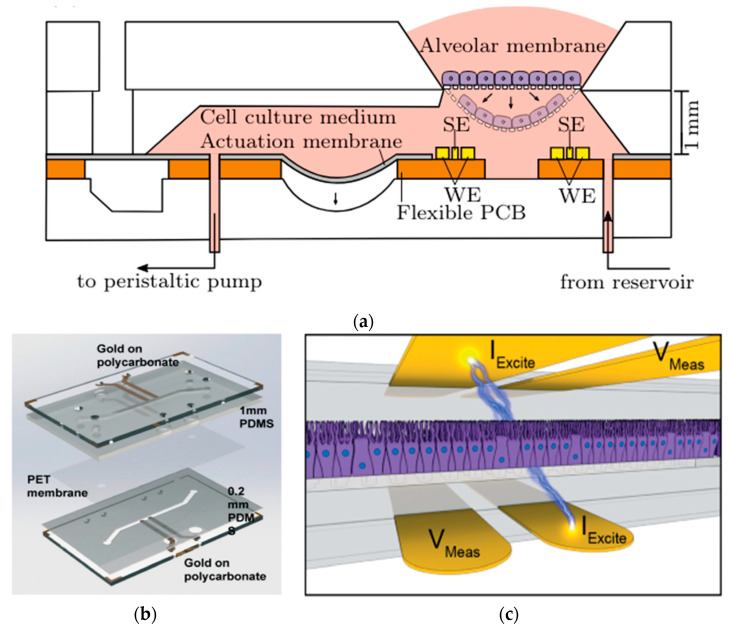
In (**a**) Cross-sectional view of the micro-impedance tomography (MITO) system incorporated into a lung-on-a-chip with sensing electrodes (SE) and the working electrodes (WE) of the MITO are positioned 1 mm beneath the cultured membrane of cells [67]; in (**b**) Assembly of human airway-on-a-chip model combining PDMS stamps, PET membrane, and polycarbonate substrates integrating TEER biosensors; and in (**c**) Illustrative view of the 4-point electrodes on the opposite sides of the cell culture with current applied between two electrodes (I_Excite_) and voltage drop measured between a second pair of electrodes (V_meas_), allowing precise TEER measurement [96]. Adapted with permission from references [67,96].

In a series of studies exploring the transformative uses of lung-on-a-chip technology, distinct PDMS microdevices with integrated sensors are highlighted. Mermoud et al. advanced a micro-impedance tomography (MITO) system incorporated into a lung-on-a-chip model that simulates breathing movements. MITO utilizes impedimetric coplanar electrodes to track both electrochemical and mechanical variations in the lung alveolar [67]. As shown in Figure 12a, this system employs a tetrapolar configuration of electrodes positioned 1 mm beneath the cell culture membrane, enabling sensitive detection of cell membrane barrier deflections without the need for apical electrodes. This approach is particularly advantageous when compared to traditional methods since it provides a straightforward and cost-effective method for real-time monitoring of lung microenvironments. However, further exploration of its sensitivity to deeper cellular processes is needed to enhance its applicability [67].

In addition, Henry et al. developed a human airway-on-a-chip with embedded TEER biosensors to monitor transepithelial electrical resistance [96]. This PDMS-based microfluidic device used a polycarbonate substrate for high optical clarity and cell culture compatibility, integrating four electrodes into the system for real-time monitoring. The TEER chip was assembled through a layer-by-layer approach, combining PDMS stamps and polycarbonate substrates treated with oxygen plasma and aqueous solutions of (3-Glycidyloxypropyl) trimethoxysilane (GLYMO) and 3-aminopropyl triethoxysilane (APTES) for optimal bonding Figure 12b. The device, featuring a porous PET membrane aligned with the PDMS layers, successfully maintained cell cultures for 62 days, including 56 days at the air-liquid interface, without cell toxicity. Compared to the previous MITO system, this study focuses more on long-term cell viability and TEER stability, making it ideal for chronic exposure studies. Its durability, however, may limit flexibility in dynamic applications [96]. Driven by cyclic mechanical strain, another study by Huh et al. focuses on reconstituting organ-level lung functions, emphasizing its ability to reproduce integrated responses to bacterial and inflammatory stimuli. This PDMS lung-on-a-chip model highlights its effectiveness in revealing toxic and inflammatory reactions to silica nanoparticles. This model excels in replicating immune responses critical for understanding disease progression, drug screening, and toxicology. However, it is less suited for structural or mechanical monitoring [97].

Furthermore, Huh et al. utilized optical and strain sensors in a PDMS microdevice to serve as a human disease model, replicating pulmonary edema caused by drug toxicity via simulating organ-level lung functions. Mimicking the alveolar-capillary interface and subjected to fluid flow and cyclic mechanical strain, the microdevice successfully reproduces the pulmonary edema found in cancer patients undergoing interleukin-2 (IL-2) treatment. Through real-time imaging and quantitative analysis facilitated by the integrated optical sensors, the study unveils the critical role of physiological breathing motions that exacerbate IL-2 toxicity, resulting in pulmonary edema [98]. This system, compared to others, uniquely combines mechanical strain with optical imaging to visualize dynamic changes, providing a holistic view of disease mechanics. However, its complexity might limit widespread adoption.

Using advanced biosensors, these studies significantly enhance the model’s capabilities by providing precise measurements of electrochemical changes, mechanical strain, and transepithelial electrical resistance. These integrated features enhance the effectiveness of lung-on-a-chip technology in replicating physiological conditions and clarifying organ-level responses. These developments highlight the adaptability and potential of lung-on-a-chip systems for disease modeling and swift diagnostics, opening up new avenues for future advancements in personalized medicine and therapeutic approaches.

#### 4.1.2. Liver-on-a-Chip

**Figure 13:**
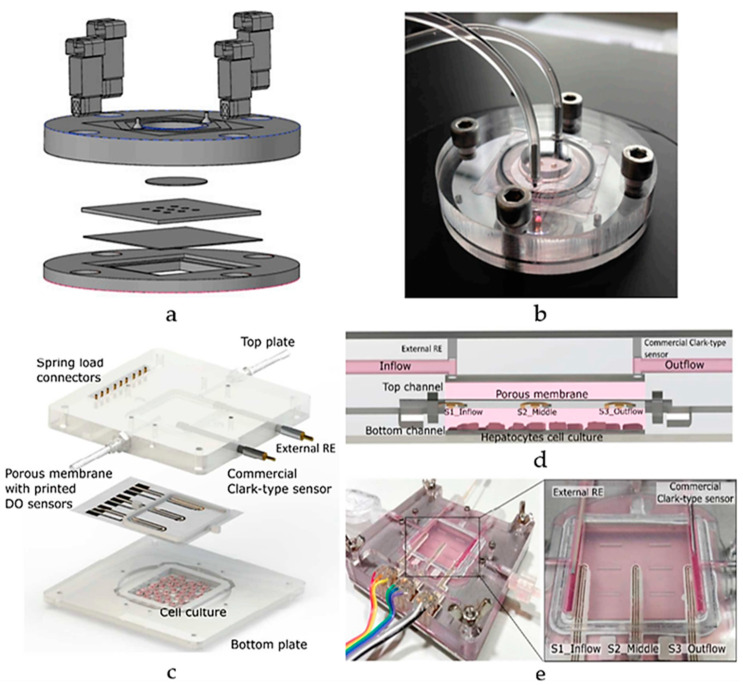
A schematic view of a liver-on-chip microfluidic platform (**a**) layers and (**b**) assembly, using a PMMA bioreactor with PDMS microwell inserts for glucose metabolism and mitochondrial function [99]; (**c**) Schematic diagram of ExoLiver integrating three electrochemical dissolved oxygen (DO) sensors; (**d**) Cross-sectional view of the system featuring the three sensors embedded within the porous membrane. (**e**) An illustration of the system assembly [100]. Adapted with permission from references [99,100].

Research on liver-on-a-chip models, employing PDMS technology, aims to enhance drug development by replicating essential liver functions crucial for drug metabolism and detoxification. Electrochemical biosensors are predominantly used in these models due to the precise measurement of biochemical changes and metabolic functions, making them ideal for replicating the complex processes of the liver. Bavli et al. created a liver-on-chip microfluidic platform using a bioreactor based on PMMA with PDMS microwell inserts to analyze glucose metabolism and mitochondrial function, incorporating automated microfluidic analysis, as illustrated in Figure 13a,b [99]. The system utilized growth-arrested HepG2/C3A cells, a type of human liver carcinoma cell, maintained under physiological conditions. The platform integrates off-chip amperometric electrochemical glucose and lactate sensors and optical oxygen sensors, allowing continuous measurements for over 24 h and 28 days, respectively. This setup revealed metabolic shifts during drug exposure (rotenone and troglitazone), with troglitazone inducing a switch to glycolysis and maintaining ATP levels, while rotenone reduced glucose uptake and increased glutaminolysis [99]. This real-time monitoring capability highlighted mitochondrial dysfunction at drug concentrations previously deemed safe, demonstrating the platform’s potential in preclinical drug development. Nonetheless, its reliance on carcinoma-derived cells, limits its relevance for normal liver studies.

Additionally, Moya et al. introduced a modular bioreactor (ExoLiver) equipped with inkjet-printed electrochemical sensors to track oxygen levels across different zones of the hepatic culture chamber [100]. As illustrated in Figure 13c,d, this system integrates three electrochemical dissolved oxygen (DO) detectors that are inkjet-printed within a fluidic channel of a very thin, porous cell culture membrane. These sensors operate on an amperometric principle, allowing real-time, in-situ observation of oxygen concentrations. The cells used in this study include primary human and rat hepatocytes. The main findings reveal that the printed sensors can detect oxygen gradients of up to 32.5% and 17.5% for human hepatocytes and rat hepatocytes, respectively, crucial for liver zonation. This approach highlights the potential of inkjet printing technology for integrating sensors into OoC devices, enabling precise, real-time monitoring of cellular respiration and metabolic functions [100]. Though its focus on specific hepatic zones may limit its broader application for whole-liver function studies. As the field progresses, engineered human liver platforms are anticipated to play a vital role in personalized drug metabolism studies, offering reliable and physiologically relevant models for preclinical drug development [101].

#### 4.1.3. Heart-on-a-Chip

The significance of Heart-on-a-chip technology lies in its transformative impact on cardiovascular research and drug development, offering a sophisticated platform to closely mimic the intricate physiology and complexities of the human heart [102]. This approach not only enhances the accuracy of studying the cardiac function and disease responses but also addresses the challenges posed by the intricate hierarchical structure of the native heart, paving the way for more precise drug screening, insights into personalized medicine, reduced reliance on animal models, and comprehensive systemic studies when integrated with other organ models. Addressing challenges in creating cardiac tissues and organ models, Shin et al. introduced an advanced aptamer-based electrochemical biosensor integrated into a microfluidic device crafted with PDMS technology, incorporating channels replicated from SU-8 photoresist masters and a thin PDMS membrane designed to detect cardiac injury biomarkers at low concentrations [103]. Functionalized with specific aptamers targeting creatine kinase-MB (CK-MB), a cardiac biomarker, this biosensor exhibits heightened sensitivity and durability compared to antibody-based sensors. Validated with human embryonic stem cell-derived cardiomyocytes (ESC-CMs), the system demonstrated dose-dependent responses to drug-induced insults, linking CK-MB secretion levels to variations in organoid cell viability and beating rates. Although this system may be less suited for long-term structural assessments, the integrated platform shows potential for continuous, sensitive biomarker monitoring in OoC systems, promising applications in drug screening and disease modeling [103].

**Figure 14:**
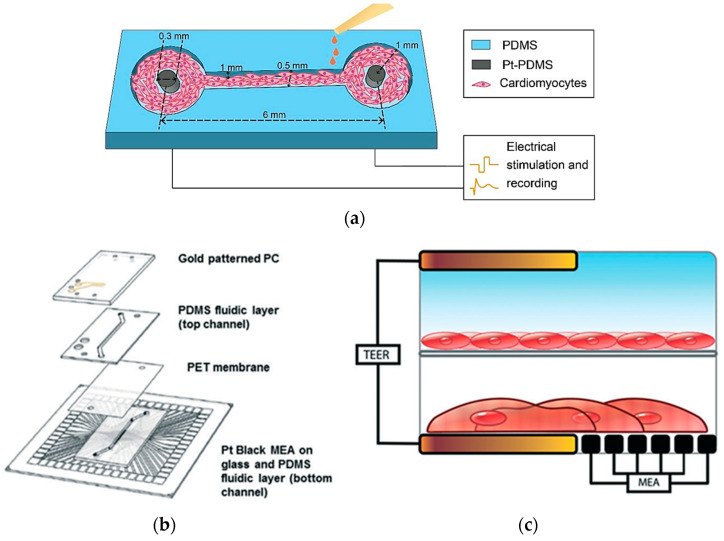
In (**a**) A schematic illustration of a PDMS-based heart-on-a-chip model with integrated platinum (Pt)-PDMS pillar electrodes [104]; (**b**) Device layers of heart-on-a-chip integrating TEER-MEA sensors; (**c**) Cross-sections of the setup with endothelial cells and cardiomyocytes cultured between two TEER electrodes [105]. Adapted with permission from references [104,105].

In heart-on-a-chip technology, electrical sensors play a pivotal role in monitoring and influencing cardiac activity, making them essential for drug testing and physiological research. Zhang et al. created a PDMS channel embedded with platinum (Pt)-PDMS pillar electrodes to cultivate 3D cardiac microtissues, employing sustained electrical stimulation and local field potential recordings of cardiomyocytes (Figure 14a). The study evaluated the effects of isoproterenol drug on contraction intervals and beating rates, demonstrating clinical relevance in drug testing scenarios. Although it offers an advantage over static systems, it is less comprehensive for modeling multi-tissue interactions [104].

Additionally, Maoz et al. established a dual-channel PDMS system that integrates endothelial cells and cardiac monolayers separated by a PET membrane [105]. Figure 14b shows that the platform includes a TEER sensor to assess endothelial monolayer integrity and Multi-Electrode Array technology to monitor cardiac electrical activity. Validated with isoproterenol tests under varying endothelial conditions, the model mimics systemic vascular drug delivery while concurrently monitoring cardiac function, though the complexity may limit scalability [105]. Moreover, Schmid et al. contributed to this field by developing a PDMS hanging drop network that enables continuous perfusion of cardiac spheroids while employing Electrical Impedance Spectroscopy (EIS) to correlate electrical spikes with tissue contractions [106]. A similar study by Bürgel et al. further enhanced cardiac spheroid studies with a tilting, high-throughput PDMS device equipped with Automated Multiplexed Electrical Impedance Spectroscopy (AMEIS), revealing insights into contraction and relaxation dynamics [107]. Incorporating these advancements in heart-on-a-chip technology, mainly through the use of biosensors, not only enhances our ability to monitor and influence cardiac activity for drug testing and physiological research but also holds promise for advancing personalized medicine by providing tailored insights into individual responses to therapeutic interventions. Future efforts should prioritize multi-tissue integration, scalability, and enhanced physiological accuracy to better replicate native heart conditions.

#### 4.1.4. Brain-on-a-Chip

The blood-brain barrier (BBB) plays a pivotal role as the guardian, separating the central nervous system from rest body, regulating the entry of essential nutrients, and barring potentially harmful substances [108]. Precise in vitro simulation of the BBB is crucial for comprehending its formation and functionality and assessing the penetration of drugs and toxins. Previous models often fell short in representing all relevant cell types and lacked the necessary flow-induced shear forces for robust tight junction development [109]. Overcoming these challenges is essential for advancing drug development targeting the brain, as high-fidelity in vitro BBB models enable effective early screening of potential therapeutic candidates. The impermeability of the BBB to many chemical compounds presents a unique hurdle in developing neuroprotective drugs, emphasizing the significance of improved in vitro models for studying and addressing neurological disorders [110].

**Figure 15:**
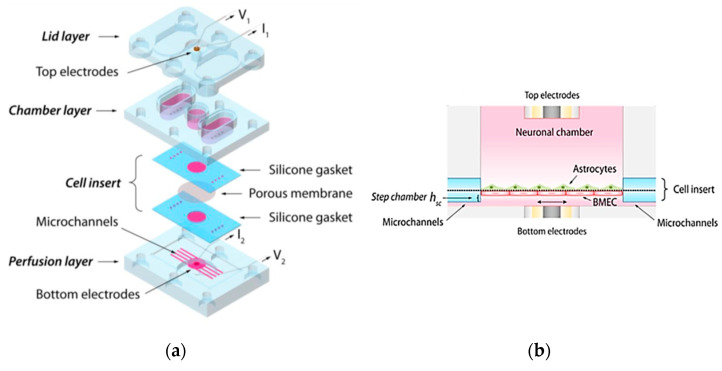

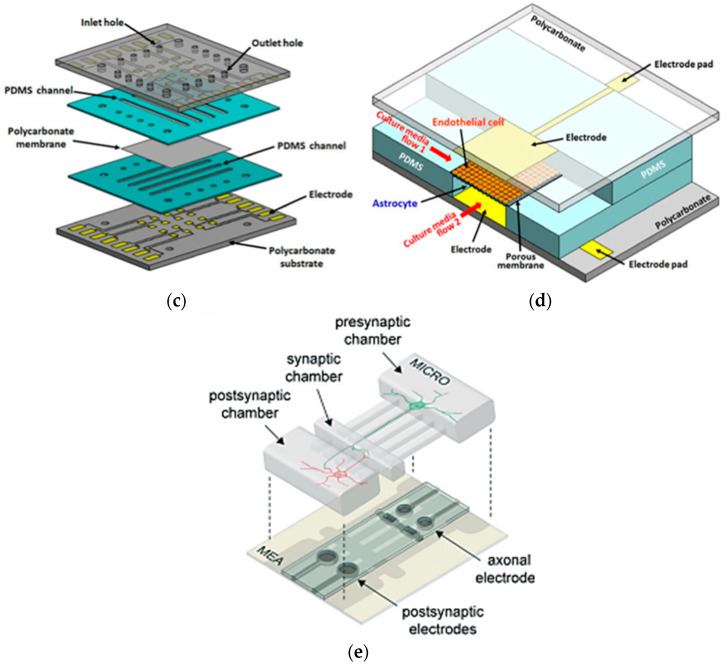
In (**a**) Design assembly of PDMS Blood-Brain Barrier (BBB) model for drug permeability screening; (**b**) Cross section view of the system integrating TEER electrodes across a co-culture of brain microvascular endothelial cells (BMECs) and rat primary [112]; (**c**,**d**) PDMS- based device incorporating TEER sensor with 16-unit chip co-culturing primary neurovascular endothelial cells and astrocytes [113]; (**e**) A schematic illustration of a PDMS-based microfluidic system integrating a MEA to mimic neural network responses [115]. Adapted with permission from references [112,113,115].

Electrical sensors play a crucial role in brain-on-a-chip technologies by enabling the monitoring of neuronal activity, which is pivotal for understanding brain function, drug responses, and disease mechanisms. Addressing the challenge of integrating sensors close to cellular barriers, a study by Van Der Helm et al. introduced a technique for directly measuring TEER in microfluidic OoCs [111]. This approach utilizes four electrodes inserted into separate channels across a PDMS-fabricated device. The model was validated using human hCMEC/D3 cerebral endothelial cells to mimic the BBB, demonstrating stable TEER values comparable to conventional systems. This model offers simplicity and reliability for TEER measurement. However, its lack of dynamic flow conditions may limit its ability to replicate the full complexity of the in vivo BBB function. Similarly, a microfluidic BBB model was developed utilizing a PDMS-fabricated platform with integrated electrical impedance sensors to mimic in vivo BBB characteristics and facilitate drug permeability studies. As illustrated in Figure 15a,b, the model incorporated brain microvascular endothelial cells (BMECs) derived from human induced pluripotent stem cells (hiPSCs) and co-cultured with rat main astrocytes on a porous membrane. The platform achieved sustained TEER values of more than 2000 Ω·cm^2^ for a period of up to 10 days. Compared to other approaches, this model’s use of dynamic flow and hiPSC-derived BMECs enhances its physiological relevance, though its mixed-species co-culture may introduce variability in cellular responses [112]. Additionally, Jeong et al. integrated an electrical impedance sensor array into a PDMS-fabricated device [113]. This system features 16 intersecting microchannels, each housing a co-culture of primary neurovascular endothelial cells and astrocytes separated by a permeable polycarbonate membrane. The chip allows for real-time assessment of barrier function through TEER measurement, demonstrating enhanced tight junction formation and reduced barrier permeability under in vivo shear stress levels (Figure 15c,d). However, its complexity may hinder scalability for routine applications.

Furthermore, Van de Wijdeven et al. present an advanced lab-on-chip system combining a microfluidic platform with a MEA to study brain circuit dynamics [114]. Fabricated using PDMS, the device features compartments for cell seeding and synaptic interactions, aiding the study of synaptic transmission and neuronal activity. In a similar study, Moutaux et al. integrated a 3-nodal PDMS-based microfluidic chip with MEAs for neurobiological and pathophysiological studies, as shown in Figure 15e. The system mimics neural network responses to injuries, making it a valuable tool for researching neurodegenerative diseases and central nervous system trauma, with potential applications in therapeutic intervention studies [115]. Although these two systems are uniquely suited for studying brain circuit behavior and injury response, they lack integration with vascular components. Utilizing an optical sensor, a microfluidic oxygenation device created by Mauleon et al. to mimic stroke events in mouse brain slices using ruthenium-based optical oxygen sensors. The device enables precise monitoring of intracellular calcium dynamics in hippocampal neurons [116]. This technology, integrated with standard electrophysiology tools, significantly advances studying neural responses under controlled oxygen conditions.

#### 4.1.5. Gut-on-a-Chip

**Figure 16:**
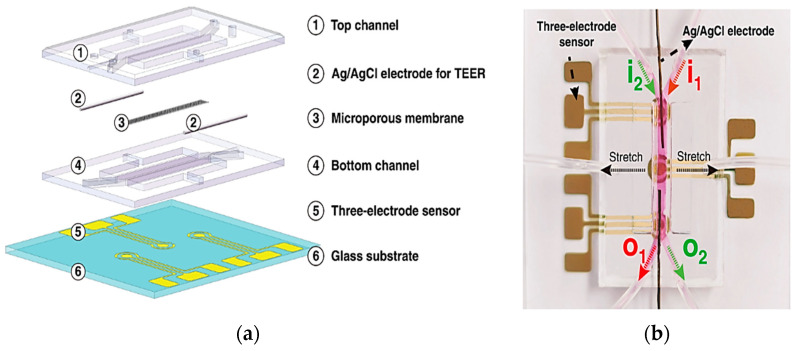
In (**a**) Schematic illustration of the combined layers of Gut-on-a-chip model; (**b**) Assembly of the device measuring the absorption of mercury undergoing mechanical strain through TEER electrodes and electrochemical sensors [118]. Adapted with permission from reference [118].

Gut-on-chip devices have investigated cellular- and tissue-level interactions in vitro. They are powerful tools for studying gut physiology, conducting drug testing and development, and exploring host-microbiome interactions [117]. Additional applications include understanding the correlations between host-microbe dynamics, human nutrition, and the microbiome. A study reported by Wang et al. integrated a gut-on-a-chip platform with TEER and electrochemical sensors to investigate mercury ion (Hg(II)) transport dynamics in human intestinal epithelium in vitro [118]. Figure 16a,b show that the PDMS chip features microchannels separated by a porous membrane, forming a tissue interface miming the intestinal barrier. Under dynamic conditions simulating physiological stress, such as fluid shear stress and cyclic mechanical strain, the chip monitored real-time Hg(II) absorption and epithelial damage. TEER measurements confirmed the formation of a functional epithelial barrier, while electrochemical sensors quantified Hg(II) absorption levels. This model is well-suited for real-time monitoring of toxicant transport and epithelial barrier function, but its application may be limited to specific toxicological studies. In addition, W. van der Helm et al. introduced an approach integrating impedance spectroscopy with electrical stimulation to assess barrier function and differentiation of human intestinal epithelial cells within gut-on-a-chip microfluidic devices [119]. The microdevice utilizes a four-electrode configuration fabricated from PC and PDMS layers, allowing precise measurement of TEER and cell layer capacitance under dynamic flow conditions. Impedance spectroscopy enabled real-time monitoring of epithelial resistance without requiring blank measurements, while electrical stimulation normalized these measurements across different chip platforms. Although this approach provides more precise and adaptable TEER measurements across platforms, it may lack the dynamic simulation of mechanical strain seen in other models. Similarly, Kim et al. integrated a TEER sensor into a ‘human gut-on-a-chip’ biomimetic microdevice to emulate the pathophysiological, mechanical, structural, transport, and absorptive characteristics of the human intestine [120]. It consists of a pair of microfluidic channels divided by a pliant membrane covered with ECM and layered with human intestinal epithelium (Caco-2) cells. This gut-on-a-chip maintained controlled fluid flow and cyclic strain to simulate peristaltic motions, supporting the growth of microbial flora while preserving human cell viability. Nonetheless, its focus on dynamic physiological conditions over advanced electrical measurement techniques, may result in reduced precision in measurement capabilities.

#### 4.1.6. Multi-Organ-on-a-Chip System

The development of multi-OoC systems is crucial for personalized medicine, offering a detailed understanding of individualized physiological responses. By replicating complex interactions between organs, these platforms enable simultaneous assessment of drug effects on various tissues, aligning with the principles of precision medicine [121]. Incorporating patient-derived cells enhances the relevance of these systems in modeling personalized disease scenarios and predicting individual responses to treatments, marking a significant stride toward more targeted and effective therapeutic development [122].

**Figure 17:**
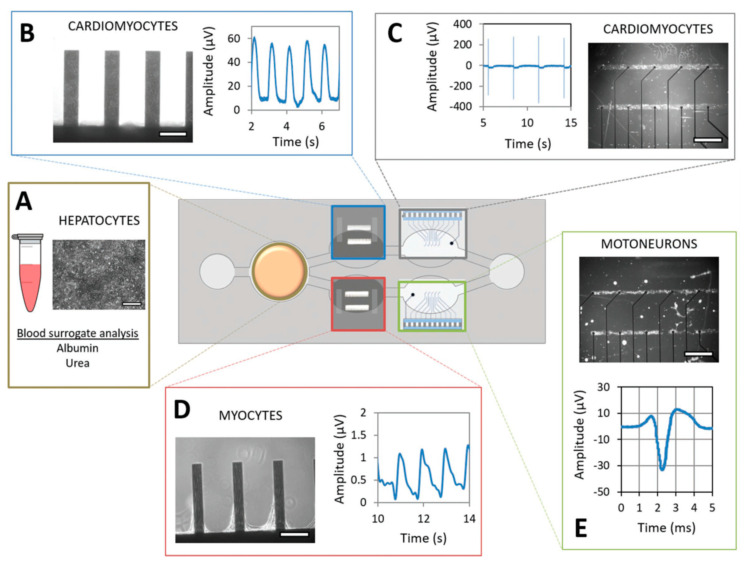
A schematic diagram of a multi-OoC system comprised of (**A**) Liver; (**B**) and (**C**) Heart; (**D**) Skeletal muscle; and (**E**) Nervous system. The 4-organ system integrates a multielectrode array and mechanical strain sensors for the measurement of neuronal and cardiac electrical assessment and skeletal and cardiac muscle contraction [123]. Adapted with permission from reference [123].

In response to the pressing need for a relevant human in vitro simulation for disease and drug studies, Oleaga et al. created a human 4-organ system to support the co-culture of skeletal muscle, liver, heart, and nervous system modules, mimicking human physiology [123]. As illustrated in Figure 17, the microfluidic chip, fabricated using PDMS, incorporated noninvasive electrical and mechanical strain sensors for real-time tracking of cellular function. Each individual chip was cultured independently until fully differentiation before being integrated into the system. The platform included electrical assessments of cardiac and neuron cells, along with mechanical measurements of cardiac and skeletal muscle contractions, but may require further exploration of real-time inter-organ signaling. This setup facilitated the in vitro evaluation of chronic toxicity, offering a promising alternative to animal testing for long-term chemical exposure studies.

**Figure 18:**
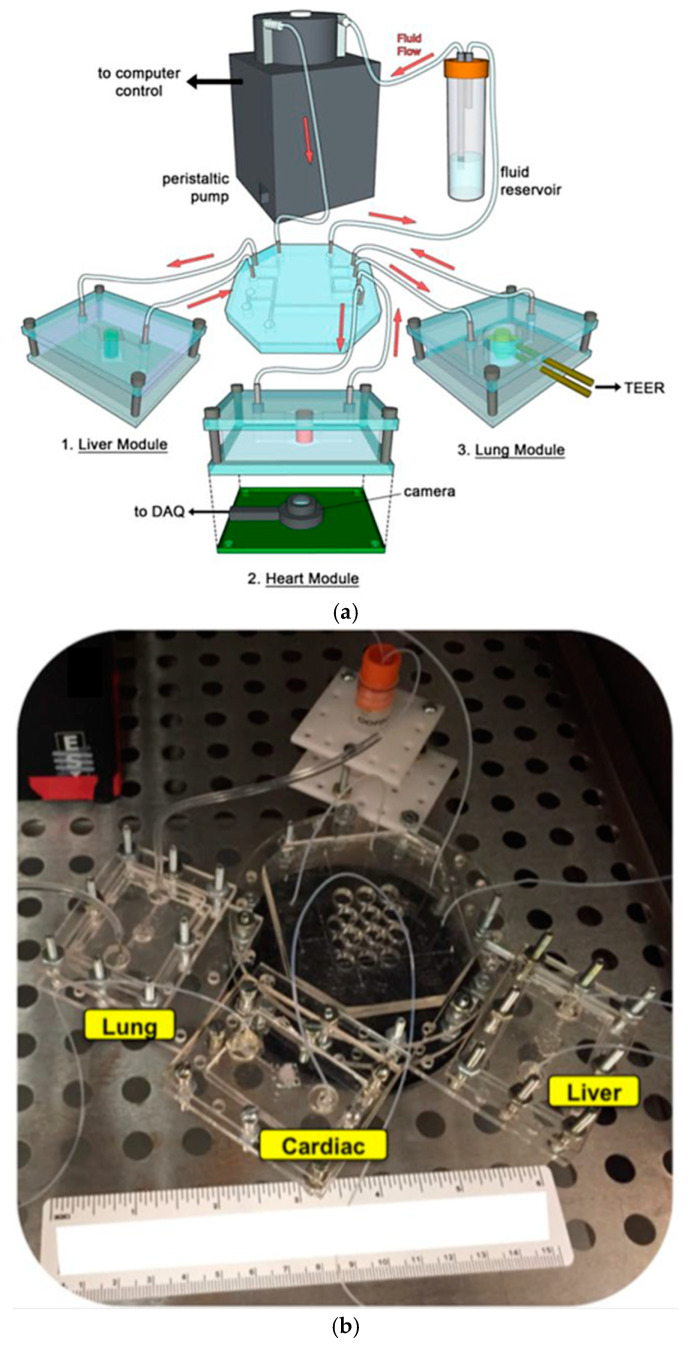
In (**a**) Design illustration of a 3-tissue multi-organ on a chip system incorporating electrochemical sensors, TEER electrodes, and an optical camera system; (**b**) The actual setup of a multi-organ system composed of lung, liver, and heart OoC devices [124]. Adapted with permission from reference [124].

**Figure 19:**
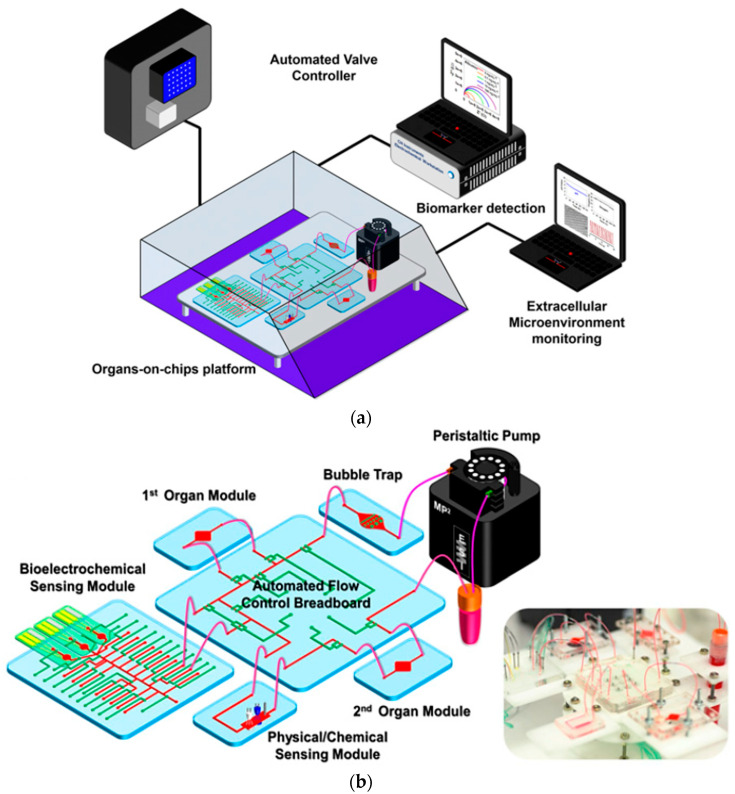
(**a**,**b**) Multi-OoC system integrating physical sensor, electrochemical sensors, and an optical sensor. A fully automated computer system controls the system [80]. Adapted with permission from reference [80].

A three-tissue OoC system was developed using advanced biosensors and PDMS for chip fabrication, integrating liver, heart, and lung organoids [124]. The liver organoids were created using main human hepatocytes, Kupffer cells, and hepatic stellate cells. Cardiac organoids included induced multipotent stem cell-derived cardiomyocytes and human main cardiac fibroblasts. Lung organoids comprised airway stromal mesenchymal cells, lung microvasculature endothelial cells, and bronchial epithelial cells. As shown in Figure 18, this integrated system includes electrochemical sensors, enabling continuous measurement of soluble biomarkers such as albumin, custom TEER electrodes incorporated into the lung module to monitor barrier function and integrity, and a real-time integrated camera system for continuous visual monitoring of the organoids, allowing the observation of cellular interactions and structural changes over time. Collectively, these sensors enhanced the analytical capabilities of the OoC platform, enabling detailed monitoring of physiological responses; however, it may face challenges in scalability due to its intricate design and diverse cell types. Similarly, Zhang et al. developed a platform for a liver-and-heart-on-a-chip system incorporating physical sensors (e.g., O2, pH, and temperature) for monitoring microenvironment parameters, miniature microscopes for observing organoid morphologies, and electrochemical sensors for soluble protein biomarkers, as shown in Figure 19. These sensors enable uninterrupted in situ monitoring, controlled via computer automation over extended periods. While this platform excels in long-term, automated monitoring, its focus on only two organs may limit its applicability [80]. These advancements underscore the versatility and possibility of multi-OoC systems in advancing drug development, disease modeling, and personalized medicine applications by closely mimicking human physiology and enabling precise control and monitoring of cellular responses and interactions.

### 4.2. Challenges of OoCs in Personalized Medicine Applications

The OoC technologies present a paradigm shift in personalized medicine with notable advantages. These platforms offer a level of precision and personalization by accurately modeling human organ functions and providing insights into individual responses to drugs and treatments [69]. The reduced dependency on animal testing is a notable ethical advancement, offering a more humane and relevant alternative for drug development. Real-time monitoring capabilities of OoC devices allow dynamic observations of cellular responses, contributing to a deeper understanding of personalized reactions to medications [125,126]. OoC systems can be tailored to replicate specific diseases, serving as valuable tools for studying individualized responses to treatments for conditions such as cancer or diabetes. Additionally, there is potential for cost efficiency in drug development processes as OoC platforms streamline testing and mitigate expenses associated with failed drug candidates and prolonged clinical trials [7].

**Figure 20:**
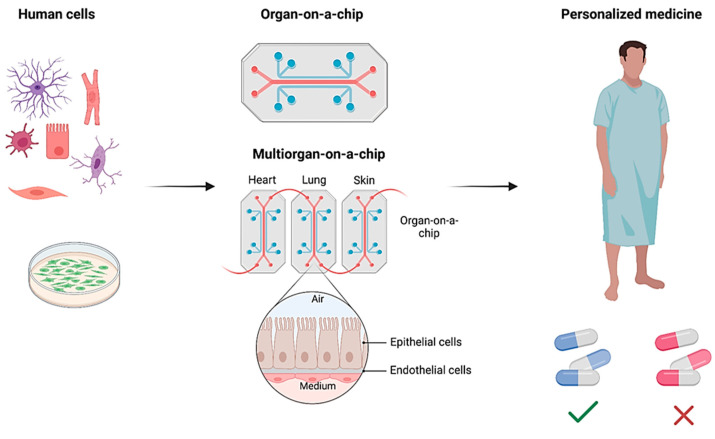
A schematic diagram of the OoC and multi-OoC for personalized medicine.

However, the adoption of OoC for personalized medicine faces several challenges. Precision medicine, aiming to customize therapies based on individual genetic and physiological factors, faces a trade-off between precision and cost within the OoC landscape [127,128]. Higher precision leads to more complex and expensive healthcare services, prompting the need for a range of precision levels to balance accessibility [10,129]. Engineered precision medicine within OoC demands high versatility, increasing regulatory complexities, and approval challenges due to diverse product lines. Our current understanding of human physiology is limited, necessitating more fundamental research within OoC to correlate biomarkers with diseases accurately [130]. Trained practitioners with genomics and engineering expertise are crucial for the successful implementation of OoC in precision medicine, requiring adaptations in medical education [131]. Privacy concerns regarding sharing genetic information and the management of big data pose ethical challenges in both precision medicine and OoC [132]. Lastly, achieving global diversity in healthcare data is essential for more inclusive precision medicine within the OoC framework [133]. Despite these hurdles, collective efforts across various fields are crucial to making precision medicine a global reality. A schematic diagram of the OoC and multi-OoC for personalized medicine is shown in Figure 20.

**Table 1:** PDMS-based Organ-on-a-chip models.

Organ-on-a-Chip	Biosensor Type	Target/Application	Advantages	Reference
Lung-on-a-chip	Electrochemical sensors—Impedimetric sensors	Pulmonary edema in cancer patients	Real-time barrier monitoring Long-term cell culture	[98]
	Electrical sensors—TEER biosensors	Monitor transepithelial electrical resistance	Real-time barrier monitoring Versatile applications	[96]
	Electrical—strain sensor	Bacterial and inflammatory stimuli	Simulates breathing motions Tracks mechanical strain	[97]
	Optical and strain sensors	Drug toxicity-induced pulmonary edema	Real-time imaging Predictive drug testing	[98]
Liver-on-a-chip	Amperometric electrochemical and optical oxygen sensors	Mitochondrial dysfunction	Precise Metabolic Monitoring Drug Effect Insights	[99]
	Amperometric electrochemical oxygen sensors	Oxygen levels across liver zonation	Real-time Oxygen Monitoring Metabolic Function Tracking	[100]
Heart-on-a-chip	Electrochemical biosensor	Detect cardiac injury biomarkers	Continuous Monitoring Enhanced Sensitivity	[103]
	Electrical sensors	Drug toxicity	Integrated Multi-functionality real-time data collection and analysis	[134]
	TEER and Multi-Electrode Array	Drug toxicity	Integrated Multi-functionality Systemic Drug Delivery Modeling	[105]
	Electrical Impedance Spectroscopy (EIS)	Toxic effects on microtissue spheroids	High-Throughput Monitoring Electrical and Mechanical Correlation	[107,108]
Brain-on-a-chip	TEER	BBB drug permeability	Real-time barrier monitoring BBB Model Validation	[111]
	Electrical impedance sensors and TEER	BBB drug permeability	Real-time barrier monitoring Tight Junction and Permeability Insights	[113,114]
	MEA	Neurobiological and pathophysiological studies	Real-time brain analysis Integrated Multi-functionality	[115,116]
Gut-on-a-chip	TEER and electrochemical sensors	Gut absorption and epithelial damage	Real-time Monitoring Dynamic Physiological Simulation	[120]
	Impedance spectroscopy with TEER sensors	Transepithelial barrier function	Real-time Impedance Monitoring Cross-platform Comparability	[119]
	TEER and strain sensor	Microbial flora studies	Integrated Multi-functionality Peristaltic Motion Simulation	[120]
	Optical sensor	Microbial flora studies	Real-time Monitoring Controlled Anaerobic Environment	[135]
Multi-organ-on-a-chip	Electrical and mechanical strain sensors	Drug screening and toxicity	Real-time Monitoring Multiorgan Integration	[123]
	Electrochemical sensors, TEER electrodes, and optical sensors	Drug screening and toxicity	Real-time barrier monitoring Integrated Visualization Automated Control	[81,125]

Abbreviations: TEER, Transendothelial Electrical Resistance; MEA, Multi-Electrode Array; EIS, Electrical Impedance Spectroscopy; BBB, Blood-Brain Barrier.

## 5. Conclusions

This comprehensive review elucidates the advancements in Polydimethylsiloxane (PDMS)-based Organ-on-a-Chip (OoC) technologies, underscoring their transformative potential in biosensors, personalized medicine, and associated biomedical applications. PDMS retains its status as a foundational material for OoC platforms owing to its intrinsic properties, which include biocompatibility, optical transparency, cost-effective fabrication, and versatility in microfluidic integration. These attributes render PDMS a favored material for engineering robust and adaptable OoC devices. Nevertheless, notable challenges persist, particularly regarding its hydrophobicity and the propensity to absorb small molecules, which may adversely affect functional performance. Surface modifications, such as plasma treatment, alongside composite blending with nanomaterials, are imperative to surmount these limitations and broaden the applicability of PDMS-based platforms. While PDMS-based devices are excellent for prototyping, they face scalability challenges in commercialization due to production limitations compared to general engineering plastics. On the other hand, we focus on four primary methods for fabricating PDMS-based microfluidic devices: photolithography, which provides high precision but requires skilled handling and specific equipment; injection molding, which is favored for its ability to produce consistent, complex structures at high volumes; hot embossing, which enables the creation of nanoscale features cost-effectively but is slower; and 3D printing, noted for its design flexibility and speed, though it faces challenges with resolution and material options.

Integrating biosensors—spanning electrochemical, electrical, and optical modalities—remains indispensable to the success of PDMS-based OoCs. These sensors afford real-time, high-resolution monitoring of cellular activities, tissue responses, and drug metabolism, providing unparalleled insights into dynamic biological systems. This review accentuates the incorporation of biosensors within specific organ models, including Lung, Liver, Heart, Brain, and Gut-on-a-Chip, as well as the emergence of multi-OoC systems. The latter enables examining systemic physiological responses and inter-organ interactions, significantly advancing complex disease modeling and personalized therapeutic strategies. Personalized medicine represents an especially promising application of PDMS-based OoCs. These platforms utilize patient-derived cells and microenvironmental mimicry to predict drug efficacy, identify potential toxicities, and devise individualized therapeutic regimens. This approach can significantly diminish reliance on animal testing, offering a more ethical and human-relevant alternative. However, widespread clinical adoption is hampered by challenges such as scalability, the necessity for regulatory standardization, and the assurance of reproducibility across diverse experimental conditions.

The future trajectory of PDMS-based OoCs resides at the confluence of advanced materials science, biosensor innovation, and state-of-the-art microfabrication techniques. Emerging technologies, including 3D printing, nanocomposite-enhanced PDMS, and modular biosensor integration, promise to address present limitations, particularly scalability and functional optimization. Furthermore, developing multi-parametric biosensors capable of monitoring complex biochemical and biophysical signals will further augment the utility of OoCs in research and clinical domains. In the context of drug discovery and toxicity assessments, PDMS-based OoCs present an ethical, cost-effective, and biologically relevant alternative to traditional animal models. Integrating artificial intelligence and machine learning methodologies allows for the extensive data generated by OoC systems to be utilized in the predictive modeling of drug responses and disease progression. Additionally, incorporating vascularized, multi-organ models can more accurately replicate human physiology, providing a holistic platform for investigating systemic diseases and the evaluation of novel therapeutics. The transition of OoC technology into routine clinical and pharmaceutical workflows holds the promise of revolutionizing diagnostics, disease modeling, and therapeutic development. PDMS-based OoCs could play a pivotal role in advancing personalized treatment strategies for cancer, rare genetic disorders, and chronic diseases. Moreover, the confluence of OoCs with point-of-care diagnostic tools may facilitate rapid and precise health monitoring, particularly in resource-constrained settings.

## References

[1] Convery N., Gadegaard N. (2019). 30 years of microfluidics. Micro Nano Eng..

[2] Azizipour N., Avazpour R., Rosenzweig D.H., Sawan M., Ajji A. (2020). Evolution of Biochip Technology: A Review from Lab-on-a-Chip to Organ-on-a-Chip. Micromachines.

[3] Capulli A.K., Tian K., Mehandru N., Bukhta A., Choudhury S.F., Suchyta M., Parker K.K. (2014). Approaching the in Vitro Clinical Trial: Engineering Organs on Chips. Lab A Chip.

[4] Zhao Y., Wang E.Y., Lai F.B.L., Cheung K., Radisic M. (2023). Organs-on-a-chip: A union of tissue engineering and microfabrication. Trends Biotechnol..

[5] Regmi S., Poudel C., Adhikari R., Luo K.Q. (2022). Applications of Microfluidics and Organ-on-a-Chip in Cancer Research. Biosensors.

[6] Ewart L., Apostolou A., Briggs S.A., Carman C.V., Chaff J.T., Heng A.R., Jadalannagari S., Janardhanan J., Jang K.-J., Joshipura S.R. (2022). Performance Assessment and Economic Analysis of a Human Liver-Chip for Predictive Toxicology. Commun. Med..

[7] Ingber D.E. (2022). Human organs-on-chips for disease modelling, drug development and personalized medicine. Nat. Rev. Genet..

[8] Huh D., Hamilton G.A., Ingber D.E. (2011). From 3d Cell Culture to Organs-on-Chips. Trends Cell Biol..

[9] Luo Y., Li X., Zhao Y., Zhong W., Xing M., Lyu G. (2023). Development of Organs-on-Chips and Their Impact on Precision Medicine and Advanced System Simulation. Pharmaceutics.

[10] Koyilot M.C., Natarajan P., Hunt C.R., Sivarajkumar S., Roy R., Joglekar S., Pandita S., Tong C.W., Marakkar S., Subramanian L. (2022). Breakthroughs and Applications of Organ-on-a-Chip Technology. Cells.

[11] Han J., Kang U., Moon E.-Y., Yoo H., Gweon B. (2022). Imaging Technologies for Microfluidic Biochips. BioChip J..

[12] Kavand H., Nasiri R., Herland A. (2022). Advanced Materials and Sensors for Microphysiological Systems: Focus on Electronic and Electrooptical Interfaces. Adv. Mater..

[13] Van J., Vries H., Firth K., Van W., Tertoolen J., Karperien J., Jonkheijm P., Denning C., IJzerman A., Mummery C. (2017). Small Molecule Absorption by PDMS in the Context of Drug Response Bioassays. Biochem. Biophys. Res. Commun..

[14] Hirama H., Satoh T., Sugiura S., Shin K., Onuki-Nagasaki R., Kanamori T., Inoue T. (2019). Glass-based Organ-on-a-chip Device for Restricting Small Molecular Absorption. J. Biosci. Bioeng..

[15] Gökaltun A., Kang Y., Yarmush M., Usta O., Asatekin A. (2019). Simple Surface Modification of Poly(dimethylsiloxane) via Surface Segregating Smart Polymers for Biomicrofluidics. Sci. Rep..

[16] Wolf M.P., Salieb-Beugelaar G.B., Hunziker P. (2018). PDMS with Designer Functionalities—Properties, Modifications Strategies, and Applications. Prog. Polym. Sci..

[17] Liu J., Yao Y., Li X., Zhang Z. (2021). Fabrication of Advanced Polydimethylsiloxane-Based Functional Materials: Bulk Modifications and Surface Functionalizations. Chem. Eng. J..

[18] Alfihed S., Bergen M.H., Holzman J.F., Foulds I.G. (2018). A Detailed Investigation on the Terahertz Absorption Characteristics of Polydimethylsiloxane (PDMS). Polymer.

[19] Kausar A. (2020). Polydimethylsiloxane-Based Nanocomposite: Present Research Scenario and Emergent Future Trends. Polym. Plast. Technol. Mater..

[20] Raj M.K., Chakraborty S. (2020). PDMS Microfluidics: A Mini Review. J. Appl. Polym. Sci..

[21] Shakeri A., Khan S., Didar T.F. (2021). Conventional and emerging strategies for the fabrication and functionalization of PDMS-based microfluidic devices. Lab A Chip.

[22] Shahriari S., Patel V., Selvaganapathy P.R. (2023). Xurography as a tool for fabrication of microfluidic devices. J. Micromech. Microeng..

[23] Ghobashy M.M., Alkhursani S.A., Alqahtani H.A., El-damhougy T.K., Madani M. (2024). Gold nanoparticles in microelectronics advancements and biomedical applications. Mater. Sci. Eng. B.

[24] Niculescu A.-G., Chircov C., Bîrcă A.C., Grumezescu A.M. (2021). Fabrication and Applications of Microfluidic Devices: A Review. Int. J. Mol. Sci..

[25] Domansky K., Sliz J., Wen N., Hinojosa C., Thompson G., Fraser J., Hamkins T., Hamilton G., Levner D., Ingber D. (2017). SEBS Elastomers for Fabrication of Microfluidic Devices with Reduced Drug Absorption by Injection Molding and Extrusion. Microfluid. Nanofluidics.

[26] Wu W.I., Rezai P., Hsu H.H., Selvaganapathy P.R., James L., Zhou Y. (2013). 1—Materials and methods for the microfabrication of microfluidic biomedical devices. Microfluidic Devices for Biomedical Applications.

[27] Jeong M., Radomski K., Lopez D., Liu J.T., Lee J.D., Lee S.J. (2024). Materials and Applications of 3D Printing Technology in Dentistry: An Overview. Dent. J..

[28] Jucius D., Lazauskas A., Grigaliūnas V., Guobienė A., Puodžiukynas L. (2020). Hot Embossing of Micro-Pyramids into Thermoset Thiol-Ene Film. Polymers.

[29] Weerakoon-Ratnayake K.M., O’Neil C.E., Uba F.I., Soper S.A. (2017). Thermoplastic nanofluidic devices for biomedical applications. Lab A Chip.

[30] Gale B.K., Jafek A.R., Lambert C.J., Goenner B.L., Moghimifam H., Nze U.C., Kamarapu S.K. (2018). A Review of Current Methods in Microfluidic Device Fabrication and Future Commercialization Prospects. Inventions.

[31] Scott S.M., Ali Z. (2021). Fabrication Methods for Microfluidic Devices: An Overview. Micromachines.

[32] Juang Y.-J., Chiu Y.-J. (2022). Fabrication of Polymer Microfluidics: An Overview. Polymers.

[33] Salentijn G.I.J., Oomen P.E., Grajewski M., Verpoorte E. (2017). Fused Deposition Modeling 3D Printing for (Bio)analytical Device Fabrication: Procedures, Materials, and Applications. Anal. Chem..

[34] Bhattacharjee N., Parra-Cabrera C., Kim Y.T., Kuo A.P., Folch A. (2018). Desktop-Stereolithography 3D-Printing of a Poly(dimethylsiloxane)-Based Material with Sylgard-184 Properties. Adv. Mater..

[35] Sun S., Fei G., Wang X., Xie M., Guo Q., Fu D., Wang Z., Wang H., Luo G., Xia H. (2021). Covalent adaptable networks of polydimethylsiloxane elastomer for selective laser sintering 3D printing. Chem. Eng. J..

[36] Weisgrab G., Ovsianikov A., Costa P.F. (2019). Functional 3D Printing for Microfluidic Chips. Adv. Mater. Technol..

[37] Yamashita T., Yasukawa K., Yunoki E. (2019). Fabrication of a Polydimethylsiloxane Fluidic Chip Using a Sacrificial Template Made by Fused Deposition Modeling 3D Printing and Application for Flow-injection Analysis. Anal. Sci..

[38] Chen C., Mehl B.T., Munshi A.S., Townsend A.D., Spence D.M., Martin R.S. (2016). 3D-printed microfluidic devices: Fabrication, advantages and limitations—A mini review. Anal. Methods.

[39] Batista Deroco P., Giarola J.d.F., Wachholz Júnior D., Arantes Lorga G., Tatsuo Kubota L., Merkoçi A. (2020). Chapter Four—Paper-Based Electrochemical Sensing Devices. Comprehensive Analytical Chemistry.

[40] Wu J., Liu H., Chen W., Ma B., Ju H. (2023). Device Integration of Electrochemical Biosensors. Nat. Rev. Bioeng..

[41] Naresh V., Lee N. (2021). A Review on Biosensors and Recent Development of Nanostructured Materials-Enabled Biosensors. Sensors.

[42] Schmidt-Speicher L.M., Länge K. (2021). Microfluidic Integration for Electrochemical Biosensor Applications. Curr. Opin. Electrochem..

[43] Fuchs S., Johansson S., Tjell A.Ø., Werr G., Mayr T., Tenje M. (2021). In-Line Analysis of Organ-on-Chip Systems with Sensors: Integration, Fabrication, Challenges, and Potential. ACS Biomater. Sci. Eng..

[44] Sabaté Del Río J., Ro J., Yoon H., Park T.-E., Cho Y.-K. (2023). Integrated Technologies for Continuous Monitoring of Organs-on-Chips: Current Challenges and Potential Solutions. Biosens. Bioelectron..

[45] Yang W., Li T., Liao S., Zhou J., Huang L. (2024). Organ-on-a-Chip Platforms Integrated with Biosensors for Precise Monitoring of the Cells and Cellular Microenvironment. TrAC Trends Anal. Chem..

[46] Grieshaber D., MacKenzie R., Vörös J., Reimhult E. (2008). Electrochemical Biosensors—Sensor Principles and Architectures. Sensors.

[47] Baranwal J., Barse B., Gatto G., Broncova G., Kumar A. (2022). Electrochemical Sensors and Their Applications: A Review. Chemosensors.

[48] Hryniewicz B., De A.G., Deller A., Bach-Toledo L., Pesqueira C., Klobukoski V., Vidotti M., Narayan R. (2023). Sensing Materials: Nanostructured Platforms Based on Conducting Polymers for Sensing. Encyclopedia of Sensors and Biosensors.

[49] Xu Q., Pan Y., Li W., Yang Z., Barhoum A., Altintas Z. (2023). 6—Amperometric Sensors. Fundamentals of Sensor Technology.

[50] Hussain C.M., Keçili R. (2020). Electrochemical Techniques for Environmental Analysis. Modern Environmental Analysis Techniques for Pollutants.

[51] Simoska O., Minteer S.D. (2021). Introduction to Electroanalytical Chemistry. Techniques in Electroanalytical Chemistry.

[52] Cordero G., Antonio E. (2018). Wearable System with Integrated Passive Microfluidics for Real-Time Electrolyte Sensing in Human Sweat. Ph.D. Thesis.

[53] Venton B.J., Cao Q. (2020). Fundamentals of Fast-Scan Cyclic Voltammetry for Dopamine Detection. Analyst.

[54] Porada R., Jedlińska K., Lipińska J., Baś B. (2020). Review—Voltammetric Sensors with Laterally Placed Working Electrodes: A Review. J. Electrochem. Soc..

[55] Antiochia R. (2022). Electrochemical Biosensors for Sars-Cov-2 Detection: Voltametric or Impedimetric Transduction?. Bioelectrochemistry.

[56] Devnani H., Sharma C., Rajendrachari S., Kenchappa K., Peramenahalli S., Vasanth S. (2023). Recent Advances in Voltammetric Sensing. Frontiers in Voltammetry.

[57] Bahadır E.B., Sezgintürk M.K. (2016). A review on impedimetric biosensors. Artif. Cells Nanomed. Biotechnol..

[58] Barhoum A., Altintas Z. (2023). Fundamentals of Sensor Technology: Principles and Novel Designs.

[59] Aydogmus H., Hu M., Ivancevic L., Frimat J.-P., van den Maagdenberg A.M., Sarro P.M., Mastrangeli M. (2023). An organ-on-chip device with integrated charge sensors and recording microelectrodes. Sci. Rep..

[60] Chen H., Luo Z., Lin X., Zhu Y., Zhao Y. (2023). Sensors-Integrated Organ-on-a-Chip for Biomedical Applications. Nano Res..

[61] Aydogmus H., Dostanić M., Jahangiri M., Sinha R., Fausto W., Mastrangeli M., Maria S.P. Fet-Based Integrated Charge Sensor for Organ-on-Chip Applications. Proceedings of the 2020 IEEE SENSORS.

[62] Clarke G.A., Hartse B.X., Niaraki Asli A.E., Taghavimehr M., Hashemi N., Abbasi Shirsavar M., Montazami R., Alimoradi N., Nasirian V., Ouedraogo L.J. (2021). Advancement of Sensor Integrated Organ-on-Chip Devices. Sensors.

[63] Morales I.A., Boghdady C.-M., Campbell B.E., Moraes C. (2022). Integrating mechanical sensor readouts into organ-on-a-chip platforms. Front. Bioeng. Biotechnol..

[64] Ferrari E., Palma C., Vesentini S., Occhetta P., Rasponi M. (2020). Integrating Biosensors in Organs-on-Chip Devices: A Perspective on Current Strategies to Monitor Microphysiological Systems. Biosensors.

[65] Spitz S., Schobesberger S., Brandauer K., Ertl P. (2023). Sensor-integrated brain-on-a-chip platforms: Improving the predictive validity in neurodegenerative research. Bioeng. Transl. Med..

[66] Rothbauer M., Bachmann B.E.M., Eilenberger C., Kratz S.R.A., Spitz S., Höll G., Ertl P. (2021). A Decade of Organs-on-a-Chip Emulating Human Physiology at the Microscale: A Critical Status Report on Progress in Toxicology and Pharmacology. Micromachines.

[67] Mermoud Y., Felder M., Stucki J.D., Stucki A.O., Guenat O.T. (2018). Microimpedance tomography system to monitor cell activity and membrane movements in a breathing lung-on-chip. Sens. Actuators B Chem..

[68] Shin H., Jeong S., Lee J.H., Sun W., Choi N., Cho I. (2021). 3D high-density microelectrode array with optical stimulation and drug delivery for investigating neural circuit dynamics. Nat. Commun..

[69] Leung C.M., De Haan P., Ronaldson-Bouchard K., Kim G.-A., Ko J., Rho H.S., Chen Z., Habibovic P., Jeon N.L., Takayama S. (2022). A guide to the organ-on-a-chip. Nat. Rev. Methods Prim..

[70] Liu H., MacQueen L.A., Usprech J.F., Maleki H., Sider K.L., Doyle M.G., Sun Y., Simmons C.A. (2018). Microdevice arrays with strain sensors for 3D mechanical stimulation and monitoring of engineered tissues. Biomaterials.

[71] Santbergen M.J.C., van der Zande M., Bouwmeester H., Nielen M.W.F. (2019). Online and in Situ Analysis of Organs-on-a-Chip. TrAC Trends Anal. Chem..

[72] Lagowala D.A., Kwon S., Sidhaye V.K., Kim D.-H. (2021). Human Microphysiological Models of Airway and Alveolar Epithelia. Am. J. Physiol. Lung Cell. Mol. Physiol..

[73] Zirath H., Spitz S., Roth D., Schellhorn T., Rothbauer M., Müller B., Walch M., Kaur J., Wörle A., Kohl Y. (2021). Bridging the Academic–industrial Gap: Application of an Oxygen and pH Sensor-Integrated Lab-on-a-Chip in Nanotoxicology. Lab A Chip.

[74] Steinegger A., Wolfbeis O.S., Borisov S.M. (2020). Optical Sensing and Imaging of pH Values: Spectroscopies, Materials, and Applications. Chem. Rev..

[75] Gruber P., Marques M.P.C., Szita N., Mayr T. (2017). Integration and Application of Optical Chemical Sensors in Microbioreactors. Lab A Chip.

[76] Ahadian S., Civitarese R., Bannerman D., Mohammadi M.H., Lu R., Wang E., Davenport Huyer L., Lai B., Zhang B., Zhao Y. (2018). Organ-On-A-Chip Platforms: A Convergence of Advanced Materials, Cells, and Microscale Technologies. Adv. Healthc. Mater..

[77] Wang X. (2014). Optical Methods for Sensing and Imaging Oxygen: Materials, Spectroscopies and Applications. Chem. Soc. Rev..

[78] Tajeddin A., Mustafaoglu N. (2021). Design and Fabrication of Organ-on-Chips: Promises and Challenges. Micromachines.

[79] Bakhchova L., Steinmann U. (2022). In-Situ Measurements of the Physiological Parameters in Lab-on-Chip Systems. TM. Tech. Mess..

[80] Zhang Y.S., Aleman J., Shin S.R., Kilic T., Kim D., Mousavi Shaegh S.A., Massa S., Riahi R., Chae S., Hu N. (2017). Multisensor-Integrated Organs-on-Chips Platform for Automated and Continual in Situ Monitoring of Organoid Behaviors. Proc. Natl. Acad. Sci. USA.

[81] Zirath H., Rothbauer M., Spitz S., Bachmann B., Jordan C., Müller B., Ehgartner J., Priglinger E., Mühleder S., Redl H. (2018). Every Breath You Take: Non-invasive Real-Time Oxygen Biosensing in Two- and Three-Dimensional Microfluidic Cell Models. Front. Physiol..

[82] Saygili E., Devamoglu U., Bayir E., Yesil-Celiktas O. (2023). An Optical pH-Sensor Integrated Microfluidic Platform Multilayered with Bacterial Cellulose and Gelatin Methacrylate to Mimic Drug-induced Lung Injury. J. Ind. Eng. Chem..

[83] Soares M.P., Flores V.F., Torre L., Fujiwara E. Online Monitoring of Cell Growth on PDMS-PDMS Reversible Microfluidic Bioreactor Integrated to Optical Fiber Sensor. Proceedings of the 2019 SBFoton International Optics and Photonics Conference (SBFoton IOPC 2019).

[84] Arshavsky-Graham S., Enders A., Ackerman S., Bahnemann J., Segal E. (2021). 3D-printed Microfluidics Integrated with Optical Nanostructured Porous Aptasensors for Protein Detection. Microchim. Acta.

[85] Alfihed S., Holzman J.F., Foulds I.G. (2020). Developments in the integration and application of terahertz spectroscopy with microfluidics. Biosens. Bioelectron..

[86] Davies K. (2010). The $1000 Genome: The Revolution in DNA Sequencing and the New Era of Personalized Medicine. Am. J. Hum. Genet..

[87] Flowers C.R., Veenstra D. (2004). The Role of Cost-Effectiveness Analysis in the Era of Pharmacogenomics. PharmacoEconomics.

[88] Pokorska-Bocci A., Stewart A., Sagoo G.S., Hall A., Kroese M., Burton H. (2014). “Personalized medicine”: What’s in a name?. Pers. Med..

[89] Shei R.-J., Paris H.L., Sogard A.S., Mickleborough T.D. (2022). Time to Move Beyond a “One-Size Fits All” Approach to Inspiratory Muscle Training. Front. Physiol..

[90] Vincent J.-L., van der Poll T., Marshall J.C. (2022). The End of “One Size Fits All” Sepsis Therapies: Toward an Individualized Approach. Biomedicines.

[91] Goetz L.H., Schork N.J. (2018). Personalized medicine: Motivation, challenges, and progress. Fertil. Steril..

[92] Yamamoto Y., Kanayama N., Nakayama Y., Matsushima N. (2022). Current Status, Issues and Future Prospects of Personalized Medicine for Each Disease. J. Pers. Med..

[93] Elemento O. (2020). The future of precision medicine: Towards a more predictive personalized medicine. Emerg. Top. Life Sci..

[94] Basak S. (2020). Unlocking the future: Converging multi-organ-on-a-chip on the current biomedical sciences. Emergent mater..

[95] Ma C., Peng Y., Li H., Chen W. (2021). Organ-on-a-Chip: A New Paradigm for Drug Development. Trends Pharmacol. Sci..

[96] Henry O.Y.F., Villenave R., Cronce M.J., Leineweber W.D., Benz M.A., Ingber D.E. (2017). Organs-on-chips with integrated electrodes for trans-epithelial electrical resistance (TEER) measurements of human epithelial barrier function. Lab A Chip.

[97] Huh D., Matthews B.D., Mammoto A., Montoya-Zavala M., Yuan Hsin H., Ingber D.E. (2010). Reconstituting Organ-Level Lung Functions on a Chip. Science.

[98] Huh D., Leslie D.C., Matthews B.D., Fraser J.P., Jurek S., Hamilton G.A., Thorneloe K.S., McAlexander M.A., Ingber D.E. (2012). A human disease model of drug toxicity-induced pulmonary edema in a lung-on-a-chip microdevice. Sci. Transl. Med..

[99] Bavli D., Prill S., Ezra E., Levy G., Cohen M., Vinken M., Vanfleteren J., Jaeger M., Nahmias Y. (2016). Real-time monitoring of metabolic function in liver-on-chip microdevices tracks the dynamics of mitochondrial dysfunction. Proc. Natl. Acad. Sci. USA.

[100] Moya A., Ortega-Ribera M., Guimerà X., Sowade E., Zea M., Illa X., Ramon E., Villa R., Gracia-Sancho J., Gabriel G. (2018). Online oxygen monitoring using integrated inkjet-printed sensors in a liver-on-a-chip system. Lab A Chip.

[101] Underhill G.H., Khetani S.R. (2018). Advances in Engineered Human Liver Platforms for Drug Metabolism Studies. Drug Metab. Dispos..

[102] Zhao Y., Rafatian N., Wang E.Y., Wu Q., Lai B.F.L., Lu R.X., Savoji H., Radisic M. (2020). Towards chamber specific heart-on-a-chip for drug testing applications. Adv. Drug Deliv. Rev..

[103] Shin S.R., Zhang Y.S., Kim D.-J., Manbohi A., Avci H., Silvestri A., Aleman J., Hu N., Kilic T., Keung W. (2016). Aptamer-Based Microfluidic Electrochemical Biosensor for Monitoring Cell-Secreted Trace Cardiac Biomarkers. Anal. Chem..

[104] Zhang N., Stauffer F., Simona B.R., Zhang F., Zhang Z.-M., Huang N.-P., Vörös J. (2018). Multifunctional 3D electrode platform for real-time in situ monitoring and stimulation of cardiac tissues. Biosens. Bioelectron..

[105] Maoz B.M., Herland A., Henry O.Y.F., Leineweber W.D., Yadid M., Doyle J., Mannix R., Kujala V.J., FitzGerald E.A., Parker K.K. (2017). Organs-on-Chips with combined multi-electrode array and transepithelial electrical resistance measurement capabilities. Lab A Chip.

[106] Schmid Y.R.F., Bürgel S.C., Misun P.M., Hierlemann A., Frey O. (2016). Electrical Impedance Spectroscopy for Microtissue Spheroid Analysis in Hanging-Drop Networks. ACS Sens..

[107] Bürgel S.C., Diener L., Frey O., Kim J.-Y., Hierlemann A. (2016). Automated, Multiplexed Electrical Impedance Spectroscopy Platform for Continuous Monitoring of Microtissue Spheroids. Anal. Chem..

[108] Lazic A., Balint V., Ninkovic D.S., Peric M., Stevanovic M. (2022). Reactive and Senescent Astroglial Phenotypes as Hallmarks of Brain Pathologies. Int. J. Mol. Sci..

[109] Brofiga M., Pisano M., Raiteri R., Massobrio P. (2021). On the road to the brain-on-a-chip: A review on strategies, methods, and applications. J. Neural Eng..

[110] Mofazzal Jahromi M.A., Abdoli A., Rahmanian M., Bardania H., Bayandori M., Moosavi Basri S.M., Kalbasi A., Aref A.R., Karimi M., Hamblin M.R. (2019). Microfluidic Brain-on-a-Chip: Perspectives for Mimicking Neural System Disorders. Mol. Neurobiol..

[111] Van Der Helm M.W., Odijk M., Frimat J.-P., Van Der Meer A.D., Eijkel J.C.T., Van Den Berg A., Segerink L.I. (2016). Direct quantification of transendothelial electrical resistance in organs-on-chips. Biosens. Bioelectron..

[112] Wang Y.I., Abaci H.E., Shuler M.L. (2017). Microfluidic blood-brain barrier model provides in vivo-like barrier properties for drug permeability screening. Biotechnol. Bioeng..

[113] Jeong S., Kim S., Buonocore J., Park J., Welsh C.J., Li J., Han A. (2018). A Three-Dimensional Arrayed Microfluidic Blood-Brain Barrier Model with Integrated Electrical Sensor Array. IEEE Trans. Biomed. Eng..

[114] van de Wijdeven R., Ramstad O.H., Valderhaug V.D., Köllensperger P., Sandvig A., Sandvig I., Halaas Ø. (2019). A novel lab-on-chip platform enabling axotomy and neuromodulation in a multi-nodal network. Biosens. Bioelectron..

[115] Moutaux E., Charlot B., Genoux A., Saudou F., Cazorla M. (2018). An integrated microfluidic/microelectrode array for the study of activity-dependent intracellular dynamics in neuronal networks. Lab A Chip.

[116] Mauleon G., Fall C.P., Eddington D.T. (2012). Precise spatial and temporal control of oxygen within in vitro brain slices via microfluidic gas channels. PLoS ONE.

[117] Ashammakhi N., Nasiri R., Barros N.R.d., Tebon P., Thakor J., Goudie M., Shamloo A., Martin M.G., Khademhosseini A. (2020). Gut-on-a-chip: Current progress and future opportunities. Biomaterials.

[118] Wang L., Han J., Su W., Li A., Zhang W., Li H., Hu H., Song W., Xu C., Chen J. (2023). Gut-on-a-chip for exploring the transport mechanism of Hg(II). Microsyst. Nanoeng..

[119] Helm M.W.v.d., Henry O.Y.F., Bein A., Hamkins-Indik T., Cronce M.J., Leineweber W.D., Odijk M., Meer A.D.v.d., Eijkel J.C.T., Ingber D.E. (2019). Non-invasive sensing of transepithelial barrier function and tissue differentiation in organs-on-chips using impedance spectroscopy. Lab A Chip.

[120] Kim H.J., Huh D., Hamilton G., Ingber D.E. (2012). Human gut-on-a-chip inhabited by microbial flora that experiences intestinal peristalsis-like motions and flow. Lab A Chip.

[121] Berg A.V., Mummery C., Passier R., Meer A.D. (2019). Personalised organs-on-chips: Functional testing for precision medicine. Lab A Chip.

[122] Sung J.H. (2021). Multi-organ-on-a-chip for pharmacokinetics and toxicokinetic study of drugs. Expert. Opin. Drug Metab. Toxicol..

[123] Oleaga C., Lavado A., Riu A., Rothemund S., Carmona-Moran C.A., Persaud K., Yurko A., Lear J., Narasimhan N.S., Long C.J. (2019). Long-Term Electrical and Mechanical Function Monitoring of a Human-on-a-Chip System. Adv. Funct. Mater..

[124] Skardal A., Murphy S.V., Devarasetty M., Mead I., Kang H.-W., Seol Y.-J., Shrike Zhang Y., Shin S.-R., Zhao L., Aleman J. (2017). Multi-tissue interactions in an integrated three-tissue organ-on-a-chip platform. Sci. Rep..

[125] Ahmed T. (2022). Organ-on-a-chip microengineering for bio-mimicking disease models and revolutionizing drug discovery. Biosens. Bioelectron. X.

[126] Sosa-Hernández J.E., Villalba-Rodríguez A.M., Romero-Castillo K.D., Aguilar-Aguila-Isaías M.A., García-Reyes I.E., Hernández-Antonio A., Ahmed I., Sharma A., Parra-Saldívar R., Iqbal H.M.N. (2018). Organs-on-a-Chip Module: A Review from the Development and Applications Perspective. Micromachines.

[127] Low L.A., Mummery C., Berridge B.R., Austin C.P., Tagle D.A. (2021). Organs-on-chips: Into the next decade. Nat. Rev. Drug Discov..

[128] Guenat O.T., Geiser T., Berthiaume F. (2020). Clinically Relevant Tissue Scale Responses as New Readouts from Organs-on-a-Chip for Precision Medicine. Annu. Rev. Anal. Chem..

[129] Ronaldson-Bouchard K., Vunjak-Novakovic G. (2018). Organs-on-a-Chip: A Fast Track for Engineered Human Tissues in Drug Development. Cell Stem Cell.

[130] van Gool A.J., Bietrix F., Caldenhoven E., Zatloukal K., Scherer A., Litton J.-E., Meijer G., Blomberg N., Smith A., Mons B. (2017). Bridging the translational innovation gap through good biomarker practice. Nat. Rev. Drug Discov..

[131] Moruzzi A., Shroff T., Keller S., Loskill P., Cipriano M. (2023). Training the Next Generation of Researchers in the Organ-on-Chip Field. Educ. Sci..

[132] Thakar R.G., Fenton K.N. (2023). Bioethical implications of organ-on-a-chip on modernizing drug development. Artif. Organs.

[133] AVCI H., GÜZEL F., EROL S., AKPEK A. (2018). Recent advances in organ-on-a-chip technologies and future challenges: A review. Turk. J. Chem..

[134] Grosberg A., Alford P.W., McCain M.L., Parker K.K. (2011). Ensembles of engineered cardiac tissues for physiological and pharmacological study: Heart on a chip. Lab A Chip.

[135] Jalili-Firoozinezhad S., Gazzaniga F.S., Calamari E.L., Camacho D.M., Fadel C.W., Bein A., Swenor B., Nestor B., Cronce M.J., Tovaglieri A. (2019). A complex human gut microbiome cultured in an anaerobic intestine-on-a-chip. Nat. Biomed. Eng..

